# A thiol-rich immunomodulatory injectable hydrogel suppresses endometrial fibrosis to restore fertilization

**DOI:** 10.1126/sciadv.aef5617

**Published:** 2026-08-12

**Authors:** Mingwei Li, Runqi Zhu, Yufeng Tian, Hanqing Yu, Liu Yang, Lucy Boyi Wang, Ping Zhou, Qiang Li, Rijian Song, Wenxin Wang

**Affiliations:** ^1^ Department of Obstetrics and Gynecology, The First Affiliated Hospital of Anhui University of Science and Technology, Huainan 232001, China.; ^2^ Institute of Precision Medicine, School of Medicine, Anhui University of Science and Technology, Huainan 232001, China.; ^3^ Research and Clinical Translation Center of Gene Medicine and Tissue Engineering, School of Medicine, Anhui University of Science and Technology, Huainan 232001, China.; ^4^ The Charles Institute of Dermatology, School of Medicine, University College Dublin, Dublin D04 V1W8, Ireland.; ^5^ University Hospital Sussex NHS Foundation Trust, Brighton, UK.; ^6^ Xuzhou Medical University Affiliated Hospital, Xuzhou, Jiangsu Province, China.

## Abstract

Endometrial fibrosis disrupts the uterine microenvironment and is a major cause of intrauterine adhesion (IUA) and infertility. Postoperative barriers are commonly used after adhesiolysis, but most act as passive spacers and do not actively remodel the fibrotic inflammatory niche. Here, we report a thiol-rich immunomodulatory injectable hydrogel (TR-gel) that forms in situ through Michael addition between acrylated hyaluronic acid and thiolated hyaluronic acid. The hydrogel conformally adheres to injured endometrium, provides tissue-matched mechanics and controlled biodegradation, and retains abundant thiol groups that create a reductive microenvironment. TR-gel scavenged reactive oxygen species, suppressed nuclear factor κB activation, promoted macrophage polarization toward a proregenerative M2 phenotype, and reduced inflammatory and fibrotic signaling. In a rat IUA model, TR-gel decreased collagen deposition, promoted angiogenesis, restored glandular architecture and embryo receptivity, and improved the fertility rate from 25.0 to 62.5%. These findings provide preclinical evidence that a thiol-rich immunomodulatory hydrogel can combine physical barrier function with microenvironmental regulation to support endometrial regeneration.

## INTRODUCTION

The endometrium is a dynamic tissue composed of the functionalis and basalis layers. These layers undergo cyclic regeneration and support embryo implantation through tightly regulated stromal-epithelial cross-talk. When this physiological architecture is disrupted by uterine surgery or infection, particularly pregnancy-related dilatation and curettage, the subsequent wound-healing process becomes abnormal. This aberrant repair results in fibrosis, which ultimately leads to intrauterine adhesion (IUA) (*1*, *2*). In such conditions, the endometrium often fails to regenerate to its normal structure, resulting in a high prevalence of menstrual disorders and reduced fertility among women of reproductive age (*3*, *4*). Infertility, which is estimated to affect 9 to 18% of women of reproductive age worldwide, has endometrial injury and the consequent impairment of embryo receptivity as its major causes, according to clinical evidence (*5*).

Now, the clinical management of IUA is primarily based on hysteroscopic adhesiolysis to restore the normal uterine cavity structure. This procedure is usually followed by the placement of intrauterine devices, balloons, or conventional hydrogels as physical barriers to reduce the risk of recurrence. Estrogen therapy is often administered concurrently to promote endometrial regeneration and minimize the likelihood of readhesion (*6*, *7*). Among these strategies, injectable hydrogel–based physical barriers have been widely used after uterine surgery owing to their favorable biocompatibility, adaptable mechanical softness, and ability to uniformly cover and isolate wound sites. In comparison with rigid implants such as intrauterine devices or balloons, injectable hydrogel can conform more accurately and completely to the irregular uterine surface (*8*). This property reduces mechanical irritation, inhibits excessive fibroblast migration, prevents abnormal healing, and maintains cavity patency during the repair process (*9*).

However, current injectable hydrogel barrier materials still present several limitations. Many products are physically cross-linked or formulated as microspheres, resulting in inadequate mechanical strength and limited stability within the dynamic uterine environment (*10*, *11*). In addition, their degradation rates are often poorly aligned with the menstrual cycle, as some degrade too rapidly and lose their effectiveness, whereas others persist excessively and interfere with tissue repair (*12*). Moreover, the complex anatomical structure of the uterine cavity restricts the conformability of conventional injectable hydrogels, thereby diminishing their overall therapeutic efficacy (*13*). Most available hydrogels act only as passive physical barriers and lack the ability to regulate the uterine microenvironment. Consequently, they fail to counteract oxidative stress or modulate immune responses to prevent fibrosis and support healthy endometrial regeneration (*14*, *15*). These challenges highlight the urgent need for advanced injectable hydrogel systems that not only adapt to the complex uterine anatomy and have controlled degradation profiles but also provide reliable antiadhesion effects and actively regulate the endometrial microenvironment.

**Fig. 1.**
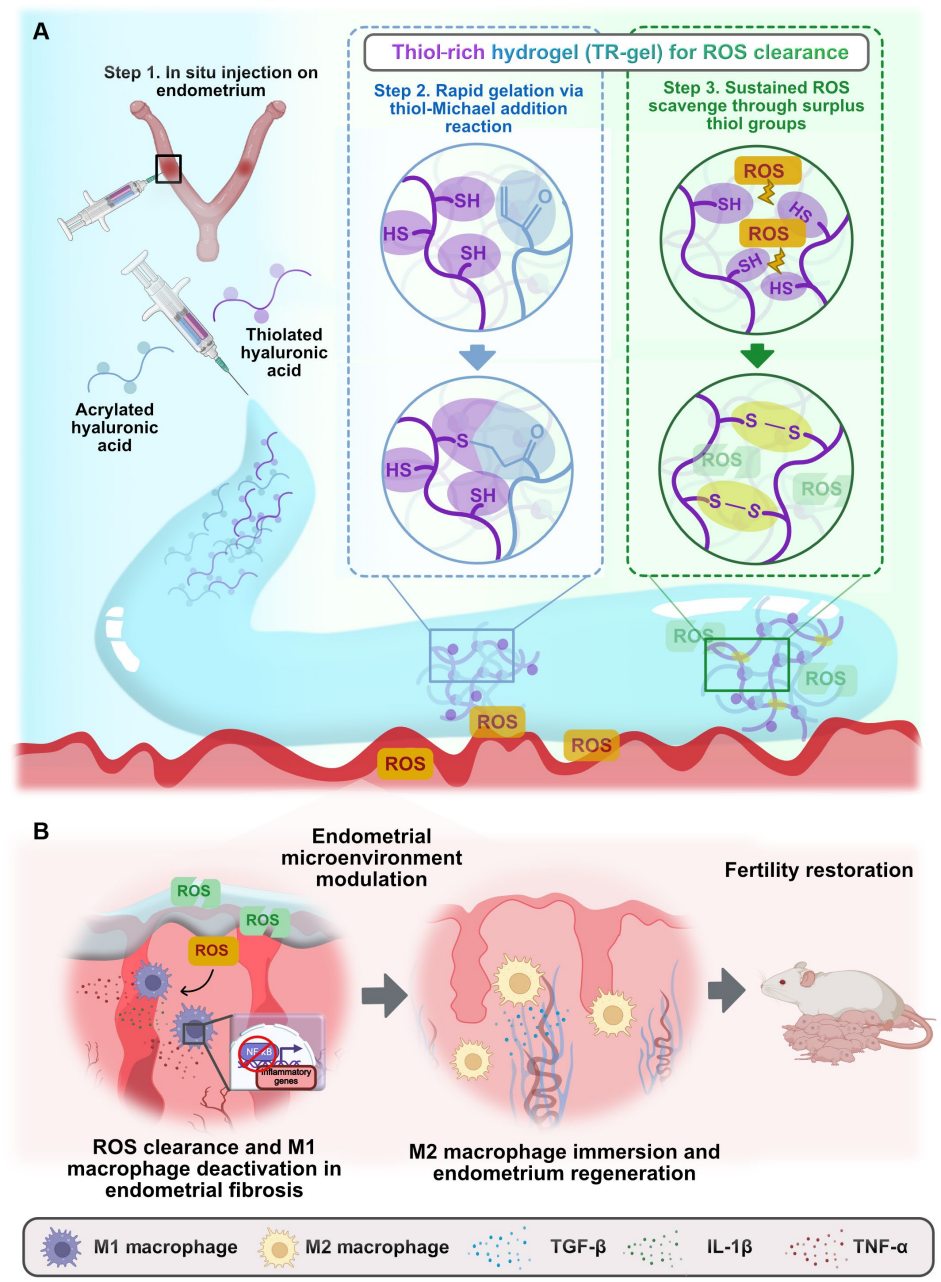
Schematic illustration of the injectable TR-gel to treat endometrial fibrosis. (**A**) Schematic of the cross-linking and ROS clearance mechanisms of thiol-rich hydrogel (TR-gel). (**B**) Mechanisms of endometrial microenvironment regulation, endometrium regeneration, and fertility restoration by TR-gel in endometrial fibrosis treatment. Created in BioRender. Milne, C. (2026) https://BioRender.com/bjyh34z.

To address these challenges, we have developed an in situ cross-linked injectable hyaluronic acid (HA)–based hydrogel via Michael addition reaction between acrylated HA (HA-A) and thiolated HA (HA-SH), as illustrated in Fig. 1. By introducing a high density of thiol groups, the resulting thiol-rich hydrogel (TR-gel) was designed not only to provide physical barrier function but also to impart antioxidant and immunomodulatory capacity. Specifically for uterine application, the TR-gel exhibits good injectability, biocompatibility, and tissue-matching mechanics, forming a stable barrier that adapts to the irregular and dynamic uterine cavity. Its controlled biodegradability (∼28 days) aligns well with the menstrual cycle, ensuring protection while avoiding prolonged foreign body retention. Beyond functioning as a physical barrier, the TR-gel efficiently scavenges reactive oxygen species (ROS), thereby suppressing oxidative stress-induced nuclear factor κB (NF-κB) activation and alleviating endometrial inflammation. This combined antioxidant and immunomodulatory activity protects local cells from ROS-mediated damage, promotes tissue regeneration, and reprograms macrophages toward the anti-inflammatory M2 phenotype, thereby mitigating excessive fibrosis and fostering favorable extracellular matrix (ECM) remodeling. In a rat IUA model, TR-gel treatment significantly restored endometrial thickness from 26.2 to 73.6% of normal levels and improved fertility rate from 25 to 62.5%. These results highlight the clinical potential of the TR-gel as an immunomodulatory injectable hydrogel system that not only effectively prevents adhesion but also promotes endometrial regeneration and restores fertility.

## RESULTS

### Preparation and characterization of the TR-gel

**Fig. 2.**
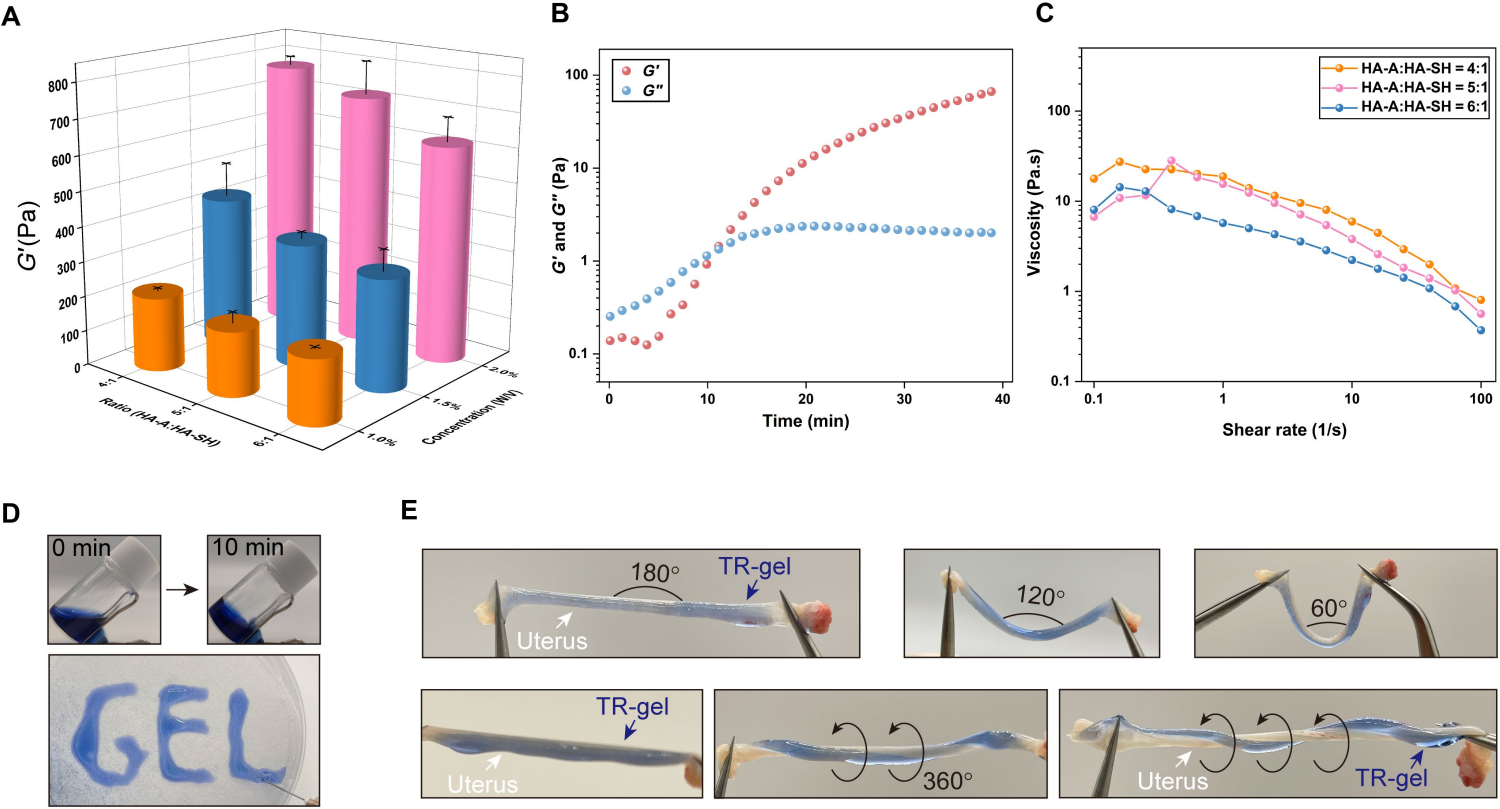
Preparation and characterization of the injectable TR-gel. (**A**) Storage modulus (*G*′) of hydrogels prepared at different HA-A:HA-SH volume ratios and total concentrations, showing the tunable mechanical properties of the system. (**B**) Rheological time-sweep curve of TR-gel (1%, 4:1 ratio), where the crossover point of *G*′ and *G*″ indicates gelation (∼10 min). (**C**) Viscosity of the HA precursor solutions at various HA-A:HA-SH ratios as a function of shear rate, confirming their injectability under shear. (**D**) Demonstration of the TR-gel’s injectability. The precursor solution, stained with Trypan blue for visualization, was smoothly extruded from a syringe to write the letters “GEL,” confirming its excellent injectability. (**E**) Ex vivo retention test of the TR-gel on rat uterine tissue. After standing for 10 min, the TR-gel maintained stable adhesion to the tissue surface under substantial mechanical challenge, including folding (60° and 120°) and twisting (360° and 720°), demonstrating good interfacial affinity and tissue retention.

The injectable hydrogels were prepared by mixing two precursor solutions, HA-A and HA-SH, at designated concentrations and volume ratios. For intrauterine application, the mechanical performance of the hydrogel is critical. Ideally, the hydrogel is required to have sufficient mechanical strength to function as an effective physical barrier against adhesion, while its viscoelastic properties should closely align with those of the endometrium to avoid notable mismatch. Notably, the apparent storage modulus (*G*′) of the nonpregnant endometrium has been reported to be ∼250 Pa (*16*). Thus, achieving suitable performance requires a balance between barrier strength and compatibility with endometrial stiffness. As shown in Fig. 2A, the results show the *G*′ of hydrogels with different concentrations and volume ratios after gelation. Increasing the polymer concentration significantly increased the hydrogel’s *G*′, whereas a higher HA-A:HA-SH volume ratio led to a decrease in the *G*′. These trends are consistent with the fact that a higher cross-linking density results in a more rigid three-dimensional (3D) hydrogel network. Specifically, the hydrogel with a 1% concentration and a 4:1 volume ratio (molar ratio of 1:1.25) had an average *G*′ of ∼210 Pa, closely matching that of the nonpregnant endometrium. Rheological time sweep analysis was used to monitor hydrogel formation, as shown in Fig. 2B. Initially, the *G*′ was smaller than the loss modulus (*G*″), indicating that the system exhibited fluid-like behavior. As cross-linking progressed, *G*′ gradually increased and intersected with *G*″, signaling the transition from a viscous liquid to an elastic solid, thus confirming hydrogel formation. The gelation time was defined as the time when *G*′ = *G*″, corresponding to the formation of a cross-linking network (*17*). The hydrogel with a volume ratio of 4:1 and a concentration of 1% reached gelation within 10 min, which is advantageous for intrauterine application by minimizing solution loss, prolonging retention, and thereby enhancing repair efficiency. Based on mechanical appropriateness and gelation capacity, a 1% formulation at an HA-A:HA-SH ratio of 4:1 was identified as an optimal candidate for subsequent evaluations. To quantify the residual free thiols after gelation, an Ellman’s assay was performed. The results confirmed that the optimal hydrogel retained a high level of free thiol groups, with a measured content of ∼1.48 mM (fig. S1), which is essential for its subsequent bioactivities. In addition, at 1 and 3 days after gelation, the unreacted thiol content in the hydrogel remained 68.9 and 47.9% of the initial level, showing the relative stability of the residual free thiols. The resulting hydrogel was designated the thiol-rich hydrogel (TR-gel).

Injectability is a key property of hydrogels intended for endometrial repair, as injectable systems must be able to distribute throughout the irregular uterine cavity, particularly into the deep recesses formed after endometrial injury (*18*). As shown in Fig. 2C, the precursor solution of TR-gel exhibited shear-thinning behavior, with viscosity decreasing as the shear rate increased. This shear-thinning behavior can be attributed to the partial disruption of intermolecular entanglements and the alignment of polymer chains under shear, thereby enabling smooth injection. As shown in Fig. 2D, the formulation was readily injected through a syringe and readily formed the letters “GEL” upon continuous extrusion before gelation, confirming its excellent injectability and suitability for in situ application.

In women of reproductive age, the endometrial glands secrete a substantial amount of mucus daily, which tends to flush out injected hydrogels from the uterine cavity. A hydrogel with good tissue retention and interfacial affinity can resist such mucus clearance, allowing it to remain in place without causing undesirable adhesion (*19*, *20*). To evaluate the tissue retention and interfacial affinity of the prepared hydrogel, an ex vivo retention test was conducted (Fig. 2E). After standing for 10 min, the excised rat endometrium was bent and subjected to twisting motions with tweezers. Under these conditions, the TR-gel remained well attached to the tissue surface, maintaining stable contact during deformation. Consequently, the TR-gel achieves a critical combination of stable retention on the lesion site and effective prevention of postoperative readhesion, both essential for IUA therapy. These observations indicate that the TR-gel has good interfacial affinity and sufficient retention capacity to remain adhered to endometrial tissue. Overall, the TR-gel exhibits favorable physical characteristics, including tunable mechanics, rapid gelation, excellent injectability, and appropriate tissue retention. These properties together enable its noninvasive delivery and stable retention within the uterine cavity, thereby enhancing therapeutic efficacy.

### Microstructure and physicochemical properties of TR-gels

**Fig. 3.**
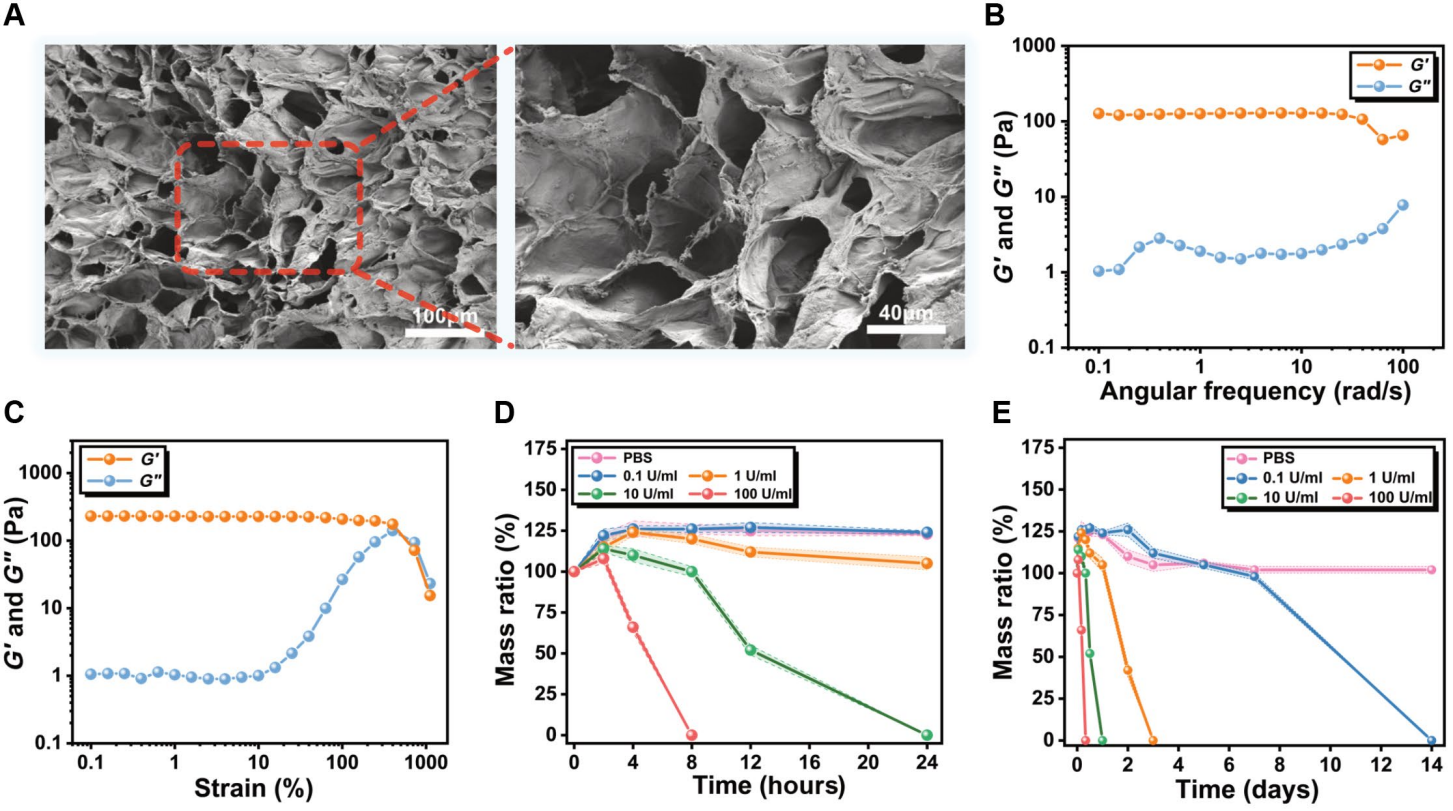
Characterization of the TR-gel. (**A**) Representative SEM images of the TR-gel at low (left) and high (right) magnification, revealing its interconnected porous microstructure. Scale bars, 100 μm (left) and 40 μm (right). (**B**) Frequency sweep of the TR-gel showing *G*′ and *G*″ under 1% oscillatory strain. (**C**) Amplitude sweep of the TR-gel showing *G*′ and *G*″ at a fixed frequency of 1 Hz. (**D**) Swelling ratio of the TR-gel in PBS and detailed degradation profile within 24 hours. (**E**) Degradation profiles of the TR-gel in PBS supplemented with the indicated concentrations of hyaluronidase at 37°C. The data on day 1 in (E) and the data at the corresponding time point in (D) were derived from the same set of samples.

We characterized the microstructure of the TR-gel using scanning electron microscopy (SEM). The TR-gel displayed a homogeneous, interconnected porous architecture, indicative of a stable 3D network (Fig. 3A). This porous network increases surface area and enhances water retention, thereby promoting intimate contact with the endometrium and reducing potential loss under mucus flow. In addition, the interconnected pores enable controlled diffusion of ROS and repair-related nutrients while restricting convective transport to preserve barrier integrity (*21*, *22*). Collectively, these structural attributes underpin the therapeutic potential of the TR-gel in the treatment of IUA.

The viscoelastic properties of the TR-gel were characterized by oscillatory rheology. *G*′ and *G*″ were measured under two regimes: a frequency sweep at 1% fixed strain and an amplitude sweep at a fixed frequency of 1 Hz. Across the tested frequency range, *G*′ consistently exceeded *G*″ (Fig. 3B), demonstrating the material’s solid-like character and robust structural stability under shear. Both *G*′ and *G*″ values fell within the range suitable for uterine tissue recovery (*23*). As strain increased, *G*′ gradually decreased while *G*″ increased; once strain exceeded ∼500%, *G*′ fell below *G*″, suggesting structural failure of the TR-gel (Fig. 3C). These findings indicate that the TR-gel maintains excellent mechanical stability under physiologically relevant deformations, which supports its stable retention and sustained barrier function in the uterine environment.

An ideal hydrogel for endometrial repair requires appropriate swelling and a controlled degradation profile, as these features provide mechanical support during the healing process while minimizing long-term foreign-body effects. The TR-gel absorbed water rapidly, reaching swelling equilibrium within 4 hours in phosphate-buffered saline (PBS) and maintaining stability thereafter (Fig. 3D). Such rapid hydration facilitates intimate contact with surrounding tissues, while the stable equilibrium prevents excessive expansion that could, otherwise, compress the regenerating endometrium. Because HA is primarily degraded by hyaluronidases, which are abundantly expressed in the peritoneum and endometrium, enzyme-dependent degradation was further evaluated. In the absence of hyaluronidase, the TR-gel retained most of its mass for more than 2 weeks, indicating intrinsic stability under nonenzymatic conditions. In contrast, increasing concentrations of hyaluronidase accelerated degradation in a dose-dependent manner, with complete dissolution occurring at ∼14 days, 3 days, 24 hours, and 8 hours at 0.1, 1, 10, and 100 U/ml, respectively (Fig. 3, D and E).

In summary, TR-gel ensures abundant free thiol groups for antioxidant and immunomodulatory functions while preserving fast gelation and mechanical appropriateness for endometrial application.

### TR-gel reshapes the endometrial regenerative niche

**Fig. 4.**
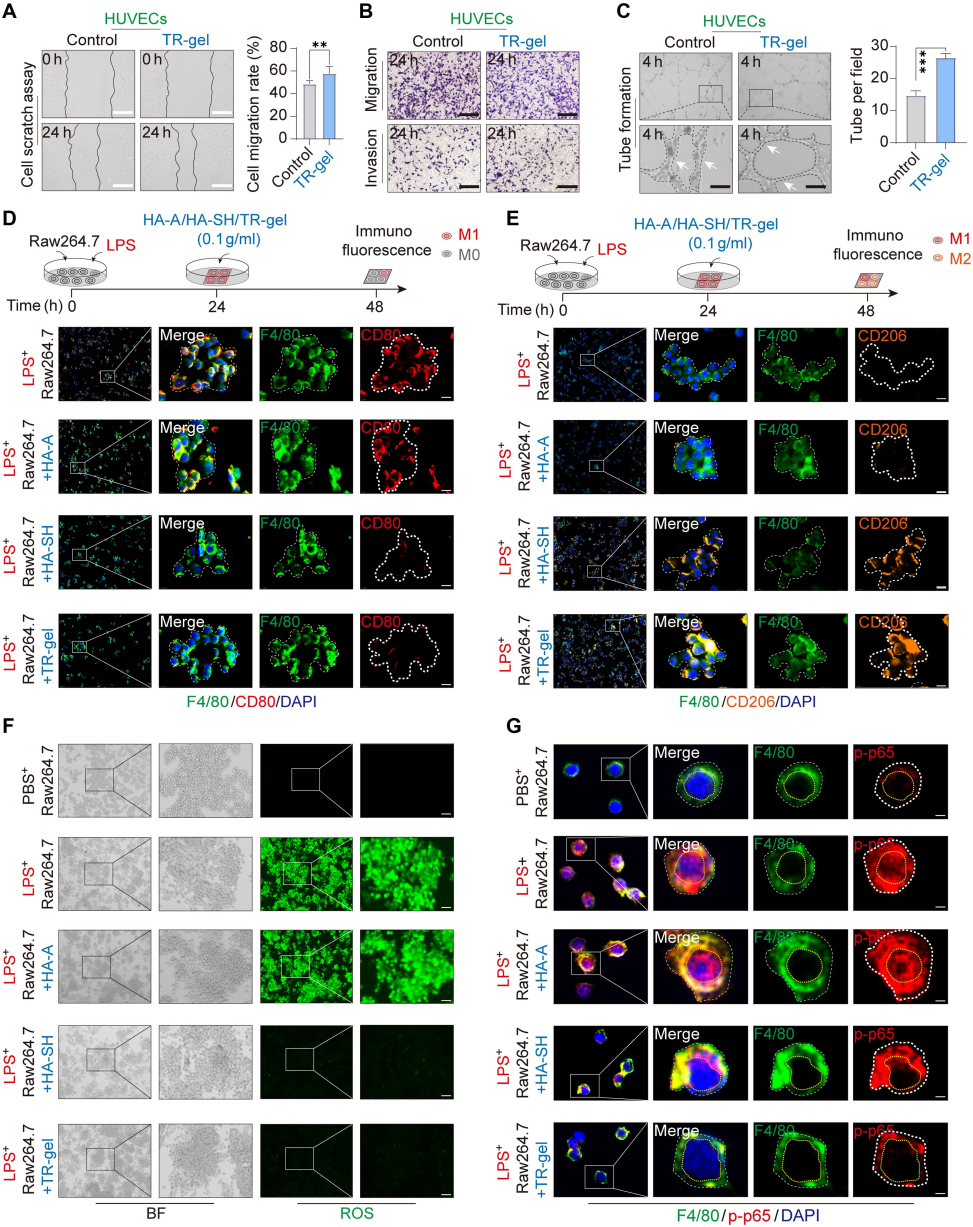
In vitro biological activity of the TR-gel. (**A**) Scratch wound-healing assay of HUVECs after 24 hours of culture with TR-gel extraction medium. Scale bars, 100 μm. (**B**) Transwell migration and invasion assays of HUVECs. Scale bar, 100 μm. (**C**) In vitro tube formation assay of HUVECs. Scale bar, 100 μm. (**D** and **E**) Immunofluorescence staining for the M1 macrophage marker CD80 and the M2 macrophage marker CD206 in RAW264.7 cells. Scale bars, 5 μm. h, hours. (**F**) Intracellular ROS levels in RAW264.7 cells, detected using a DCFH-DA fluorescent probe following treatment with LPS or LPS plus TR-gel. Scale bars, 10 μm. (**G**) Immunofluorescence analysis of NF-κB pathway activation, as indicated by phosphorylated p65 (p-p65) levels, in RAW264.7 macrophages. The yellow line indicates the nuclear area, and the white dotted line indicates the cytoplasmic area. Scale bars, 2 μm. Data are presented as means ± SD. **P < 0.01, ***P < 0.001. BF, bright field.

Because the TR-gel will contact the endometrium, its biocompatibility is crucial to its application prospects (*24*). To examine the cytotoxicity of the TR-gel, human umbilical cord endothelial cells (HUVECs) were used for in vitro cell viability tests using the Cell Counting Kit (CCK-8) assay. When HUVECs were cultured with the TR-gel extract medium, there was no significant difference in cell viability compared to the control, indicating the good cytocompatibility of the TR-gel containing abundant reductive thiols (fig. S2A). Angiogenesis plays a vital role in IUA recovery, and treatments targeting angiogenesis have proven to be efficacious for IUA. Angiogenesis is a multifaceted process comprising multiple steps. Endothelial cells undergo activation when exposed to angiogenic stimuli, leading to degradation of the basement membrane and subsequent sprouting, migration, and proliferation to create capillary tube branches that eventually join with adjacent vessels (*25*, *26*). We thus evaluated the impact of the TR-gel on key angiogenic processes in HUVECs, beyond the proliferative effect previously demonstrated. A scratch wound assay showed that the TR-gel significantly promoted HUVEC migration after 24 hours compared to the control (Fig. 4A). Similarly, Transwell migration and invasion assays confirmed the presence of a significant promigratory or proinvasive effect (Fig. 4B). In contrast, the endothelial cell tube formation assay revealed a proangiogenic effect. The TR-gel treatment significantly enhanced the total tube length per field compared to the control (Fig. 4C). In summary, the TR-gel not only enhanced HUVEC migration and invasion but also significantly promoted proliferation and tube formation. Combined with its previously demonstrated biocompatibility and proproliferative effect, these properties suggest that it can support angiogenesis, which is favorable for uterine cavity healing.

The pathogenesis of IUAs involves dysregulated inflammation and excessive fibrosis (*27*, *28*). An imbalance between proinflammatory M1 and restorative M2 macrophage polarization is a key driver of this pathological process, sustaining inflammation and promoting fibrosis (*29*, *30*). To investigate the immunomodulatory role of the TR-gel, we conducted in vitro experiments using the RAW264.7 macrophage cell line. Immunofluorescence analysis indicated that the TR-gel promoted macrophage polarization toward the M2 phenotype. It also effectively suppressed the release of proinflammatory factors from lipopolysaccharide (LPS)–induced M1 macrophages (Fig. 4, D and E). This dual action mitigates excessive inflammation during uterine repair, reduces aberrant collagen deposition, and ultimately promotes the regeneration of the functional endometrial layer.

The overproduction of ROS at wound sites induces significant oxidative stress, resulting in structural damage to essential cellular components, including proteins, lipids, and DNA. This damage not only triggers inflammation but also disrupts key healing processes, including impairing stem cell and macrophage function, suppressing angiogenesis, and causing endothelial dysfunction (*31*). These cumulative effects exacerbate tissue damage and substantially hinder the wound regeneration process (*32*). We next quantified intracellular ROS levels in cells treated with hydrogels using the 2′,7′-dichlorodihydrofluorescein diacetate (DCFH-DA) probe and confocal laser scanning microscopy (CLSM). Green fluorescence (indicating ROS) was significantly elevated in the LPS and HA-A groups but was markedly reduced in the HA-SH and TR-gel groups compared to the LPS control (Fig. 4F). These findings indicate that the TR-gel scavenges extracellular ROS, thereby reducing the oxidative burden on macrophages, alleviating cellular damage, and attenuating proinflammatory signaling.

The NF-κB signaling pathway plays a central role in inflammatory diseases, including IUA, by driving the expression of proinflammatory mediators (*33*). This pathway is also implicated in the repair of damaged endometrium (*34*). To determine whether the TR-gel inhibits the LPS-activated NF-κB pathway, we assessed the nuclear translocation of phosphorylated p65, a key subunit of the NF-κB transcription complex (Fig. 4G and fig. S3). The LPS-induced nuclear translocation was significantly suppressed by the TR-gel, while HA-SH alone also displayed a comparable suppression effect, suggesting the critical role of the thiol-rich component in the TR-gel. In summary, the TR-gel scavenges excess ROS, reduces oxidative stress, promotes macrophage repolarization, and inhibits NF-κB activation, thereby effectively controlling the inflammatory response. This anti-inflammatory microenvironment promotes the polarization of macrophages from a proinflammatory M1 to a proreparative M2 phenotype, reduces endometrial fibrosis, and lastly promotes the repair of damaged endometrium.

### Orchestrating endometrial repair via immunomodulation

Because the hydrogel will come into contact with damaged endometrial tissue and potentially be exposed to blood, a blood compatibility evaluation was performed. The hemolysis rate of the TR-gel extract was negligible and well below the 5% safety threshold (fig. S2B), indicating excellent hemocompatibility. Ensuring the biological safety of the TR-gel is crucial for its application in IUA. Thus, hematoxylin and eosin (H&E) staining of the main organs (brain, heart, liver, uterus, lung, and kidney) and serum chemistry indices were performed to evaluate biological safety (fig. S2C). The blood routine test of rats implanted with the TR-gel showed no significant change compared to the PBS group. The main liver function markers, aspartate aminotransferase and alanine aminotransferase, did not show any significant change (fig. S2D). Meanwhile, the main organs (brain, heart, liver, uterus, lung, and kidney) of rats were harvested for pathological examination 14 days after the injection of the TR-gel. H&E staining revealed no evident pathological changes in the major organs of the TR-gel group compared to the PBS group (fig. S4A). We next evaluated the in vivo degradation behavior of the TR-gel through subcutaneous injection in rats. The results showed that the gel mass underwent ∼40% degradation by postoperative day (POD) 14 and was nearly completely degraded by POD 28. This degradation profile covers the typical duration of endometrial repair (up to 21 days in the proliferative phase), thereby avoiding potential compression of endometrium caused by nondegradable hydrogels. Histological analysis of peri-implant tissues harvested on POD 14 and 28 (H&E and Masson’s staining) showed minimal inflammatory cell infiltration and no appreciable tissue damage at the implantation site. In addition, the expression levels of tumor necrosis factor–α (TNF-α) and α-SMA did not differ significantly from those in control tissues (fig. S4B). Our material demonstrates robust regenerative efficacy in repairing the endometrium, coupled with a favorable safety profile and controllable degradation behavior, which collectively address the key prerequisites for its clinical translation.

**Fig. 5.**
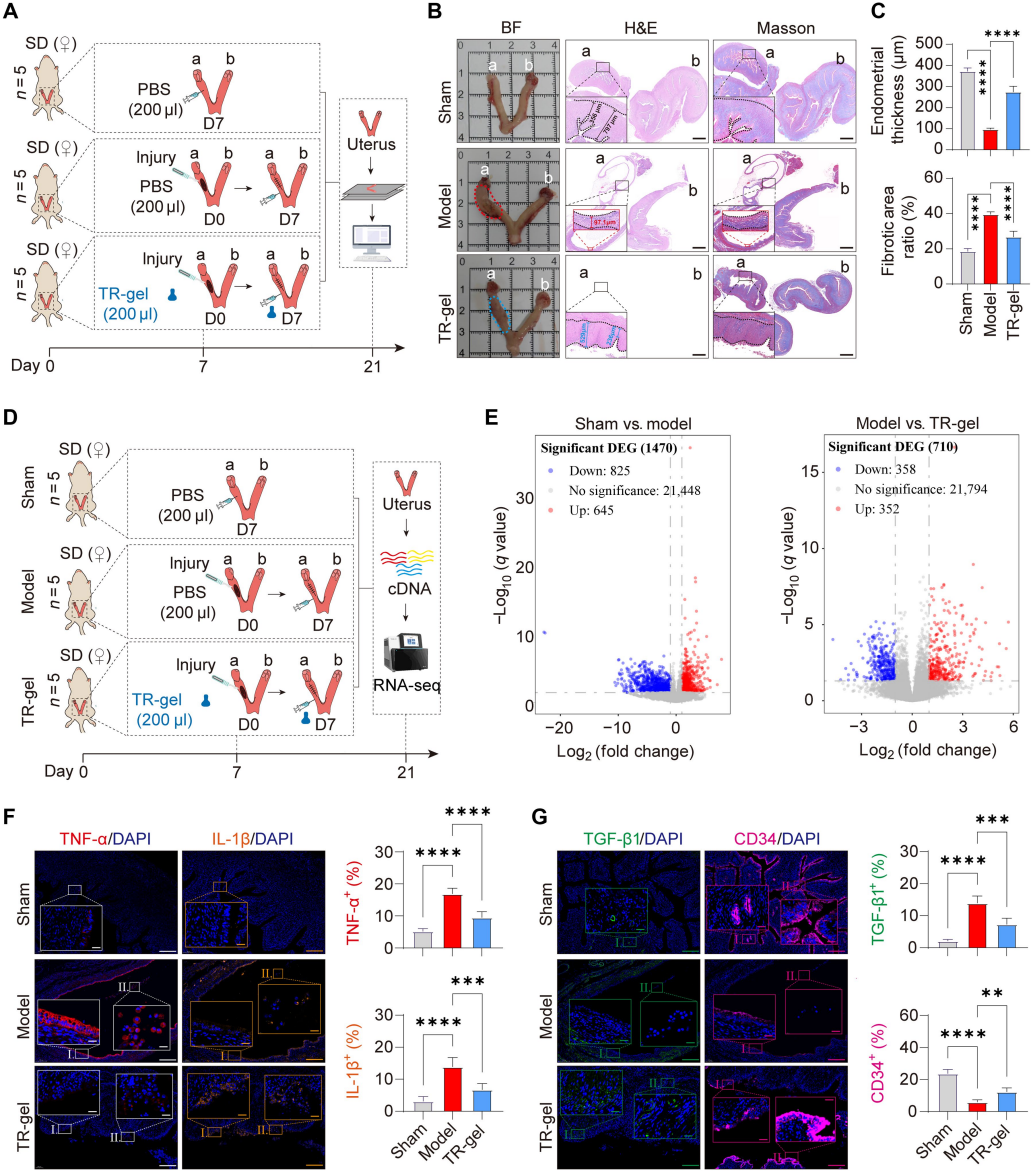
Therapeutic efficacy of the TR-gel in a rat IUA model. (**A**) Schematic of the experimental timeline and treatment protocol. (**B**) Representative gross images, H&E staining, and Masson’s trichrome staining of uterine tissues 14 days posttreatment. The magnified area shows endometrial thickness and collagen deposition. Scale bars, 500 μm. (**C**) Quantitative analysis of endometrial thickness and fibrosis area across different groups. (**D**) Workflow for the transcriptomic RNA sequencing analysis of endometrial tissues. RNA-seq, RNA sequencing. (**E**) Volcano plots displaying differentially expressed genes (DEGs) in the model versus sham comparison (left) and the TR-gel versus model comparison (right). (**F**) Immunofluorescence staining and corresponding quantification of TNF-α and IL-1β in endometrial tissues. (**G**) Immunofluorescence staining and corresponding quantification of TGF-β1 and CD34. Scale bars, 10 μm (top) and 200 μm (bottom). Data are presented as means ± SD. **P < 0.01, ***P < 0.001, and ****P < 0.0001.

Because most IUA results from endometrial injury following uterine manipulation, we established a rat endometrial curettage model to evaluate the therapeutic efficacy of the TR-gel (fig. S5). To minimize hormonal confounding, surgeries were performed during the diestrus phase, as determined by vaginal cytology (*35*). Two weeks after treatment, the rats were euthanized, and their uterine tissues were collected for gross imaging and tissue section staining (Fig. 5A). Gross examination revealed severe IUAs and fluid accumulation (hydrometra) in the model group, in contrast to the sham-operated controls. Treatment with the TR-gel markedly prevented adhesion formation, and no significant fluid accumulation was observed. Histological analysis (H&E staining) showed an intact endometrial structure with a continuous epithelial layer and prominent glands and blood vessels in the sham group. In contrast, the model group exhibited severe endometrial atrophy, characterized by significant thinning and gland loss. The TR-gel group, however, demonstrated substantial restoration of the endometrium, with increased thickness, greater gland density, and a restored epithelial layer. As the suppression of fibrosis is crucial for preventing IUAs (*36*, *37*), we next quantified collagen deposition using Masson’s trichrome staining; this method colors collagen fibers blue. The injured endometrium in the model group exhibited intense blue staining, indicating excessive collagen deposition. In contrast, TR-gel treatment markedly reduced the blue staining area, suggesting inhibition of fibrosis (Fig. 5B). The average endometrial thickness was significantly greater in the TR-gel group than in the model group, restoring it to 73.6% of the sham group level. Furthermore, the collagen area fraction was significantly lower in the TR-gel group than in the model group (Fig. 5C). To further corroborate the antifibrotic effect, we quantified the hydroxyproline (HYP) content in endometrial tissues as a biochemical indicator of collagen deposition (fig. S6A). In line with the histological observations, HYP levels were significantly reduced in the TR-gel group compared with the model group (fig. S6B), further confirming the attenuation of collagen accumulation. Together, the gross and histological evidence demonstrates that the TR-gel not only acts as a physical barrier but also actively mitigates fibrosis and promotes endometrial regeneration.

Given the demonstrated efficacy of the TR-gel in promoting uterine regeneration, we sought to elucidate its underlying mechanisms. We investigated the therapeutic mechanism of the TR-gel using RNA sequencing and immunofluorescence staining in a rat model of endometrial injury. Two weeks posttreatment, uterine tissues were harvested for transcriptome sequencing to compare global gene expression profiles across the different treatment groups (Fig. 5D). Differential expression analysis identified 1470 differentially expressed genes (DEGs) (645 up-regulated) in the model group versus the sham group. Comparing the TR-gel group to the model group revealed 710 DEGs (358 down-regulated) (Fig. 5E). Gene Ontology (GO) analysis demonstrated that the DEGs were enriched in inflammatory response terms (fig. S7, A and B). Kyoto Encyclopedia of Genes and Genomes pathway analysis revealed enrichment in leukocyte transendothelial migration and ECM-receptor interaction pathways critical for endometrial repair (fig. S7, C and D). A heatmap of secretory factor genes confirmed the activation of key processes during regeneration, including the inflammatory response (e.g., *Il1a*, *Il1b*, *Il6*, and *Tnf*), fibrosis (e.g., *Tgfb1*, *Tgfb2*, *Col1a1*, and *Acta2*), and cell proliferation/angiogenesis (e.g., *Cd80*, *Cd163*, *Vegfa*, and *Cd34*) (fig. S7E). Furthermore, proangiogenic and remodeling factors were highly expressed during regenerative stages. Collectively, our transcriptomic data indicate that the TR-gel promotes endometrial repair by coordinately modulating critical pathways: It attenuates inflammation and fibrosis while simultaneously promoting endothelial cell migration, angiogenesis, and proliferation.

Extensive cell proliferation is a crucial benchmark for tissue reconstruction, and the occurrence of neovascularization ensures optimal nutritional conditions for cell proliferation, constituting a key element in tissue regeneration (*38*). We performed immunofluorescence analysis to delineate the pathology of IUA, which is characterized by endometrial fibrosis, a chronic inflammatory process involving excessive ECM deposition that leads to endometrial dysfunction (*39*). Cytokines such as TNF-α and interleukin-1β (IL-1β) are key regulators of fibrosis, with TNF-α being a potent proinflammatory mediator positively correlated with fibrotic progression (*40*). TR-gel treatment significantly down-regulated the levels of both TNF-α and IL-1β compared to the model group (Fig. 5F). A pivotal mechanism in endometrial fibrosis is the transforming growth factor–β (TGF-β1)–mediated differentiation of stromal cells into myofibroblasts (*41*, *42*). Consistent with an antifibrotic effect, TR-gel treatment markedly down-regulated TGF-β1 expression compared to the model group (Fig. 5G). To evaluate the in vivo immunomodulatory effects of TR-gel, immunofluorescence staining was performed for F4/80 (a pan-macrophage marker), CD80 (an M1 macrophage marker), and CD206 (an M2 macrophage marker) (fig. S8). Compared with the sham group, the model group exhibited a marked increase in the infiltration level of M1-like (CD80^+^) macrophages within the injured uterine area, reaching 45.1% (fig. S8A). Notably, treatment with TR-gel significantly reduced this M1-like cell infiltration to ∼12.4%, indicating that TR-gel suppresses M1-like macrophage infiltration and thereby alleviates the inflammatory response. In contrast, the level of M2-like (CD206^+^) macrophage infiltration in the injured area showed no significant change in the model group relative to the sham group, whereas robust M2-like cell infiltration was observed in the TR-gel group (fig. S8B). Collectively, these findings suggest that TR-gel promotes the polarization of M1 toward M2 macrophages in the injured uterine region, thus attenuating inflammation and facilitating tissue repair.

Endometrial receptivity and functional repair are critically dependent on successful neovascularization, which supports the resolution of fibrosis and enables cellular proliferation necessary for restoring fertility (*42*). CD34 is mainly used to detect the presence of endothelial tissue to evaluate angiogenesis (*43*). The sham group exhibited a uniform distribution of CD34-positive vessels. This pattern was severely disrupted in the model group, which showed significantly reduced CD34 expression and damaged vascular architecture. In contrast, the TR-gel group restored a uniform network of hollow, circular vessels, with CD34 expression and morphology closely resembling the sham group (Fig. 5G). In summary, these findings demonstrate that the TR-gel promotes uterine repair by concurrently mitigating inflammation and fibrosis while restoring functional angiogenesis, thereby improving endometrial receptivity.

### TR-gel restores fertility in injured endometrium

**Fig. 6.**
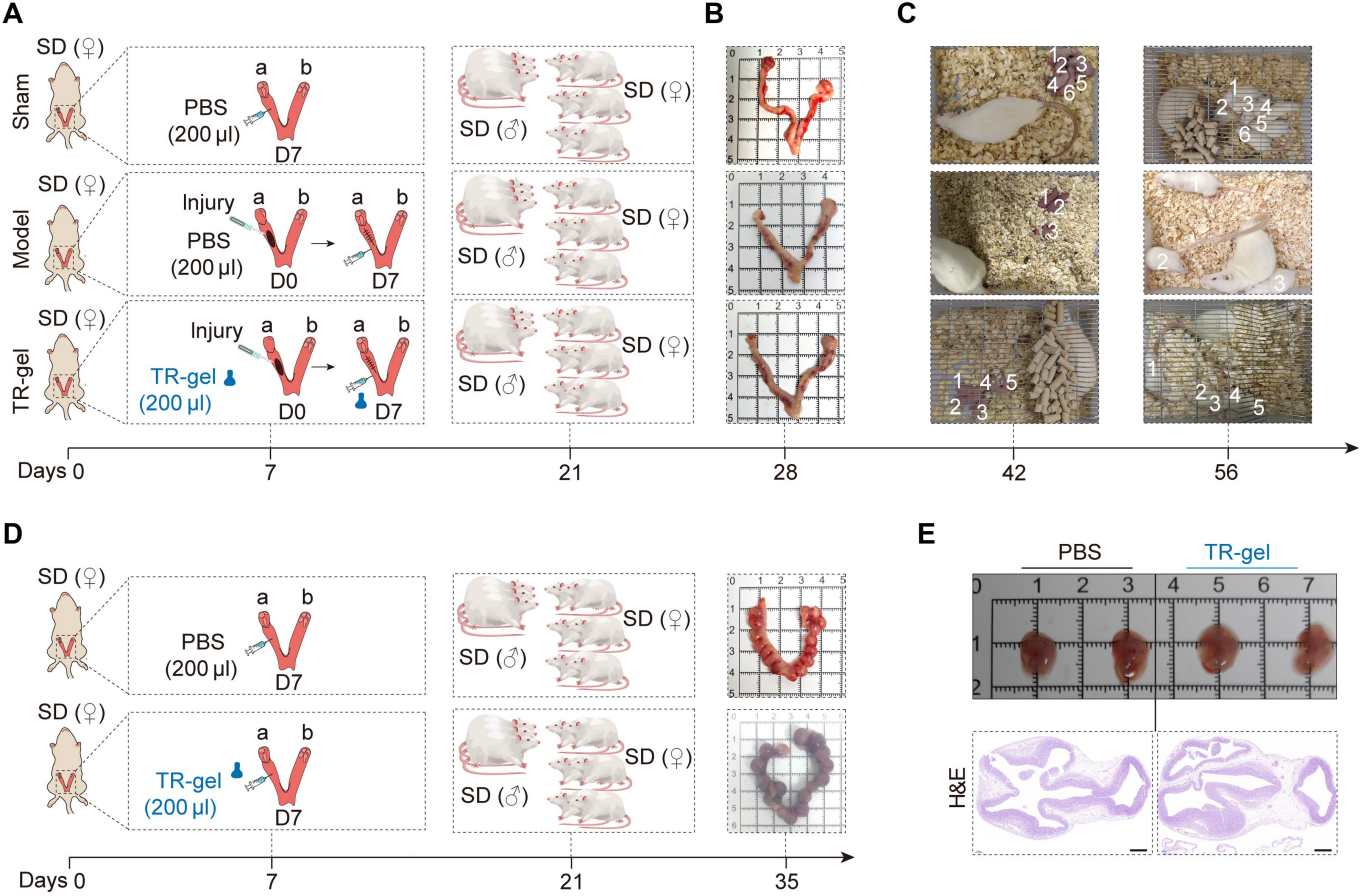
The TR-gel restores fertility in a rat model of endometrial injury. (**A**) Schematic of the experimental timeline and fertility protocol. D, day. (**B**) Embryo implantation sites in the different groups. (**C**) Postnatal development of pups from the TR-gel group at birth (postnatal day 0, P0) and on postnatal day 14 (P14). (**D**) Comparative view of uterine horns with embryos from the PBS control and TR-gel-treated groups at 14 days postcoitum (dpc). (**E**) H&E-stained cross sections of embryos from both groups at 14 days postcoitum, showing normal morphological development. Scale bars, 200 μm. SD, Sprague-Dawley.

Functional restoration of fertility is the definitive metric for assessing endometrial regeneration (*44*). To comprehensively assess the therapeutic potential of the TR-gel in endometrial repair, we performed a multistage fertility assay in a rat model of endometrial injury, examining embryo implantation, fetal development, and live birth outcomes. A within-subject experimental design was used in bicornuate uteri, with one uterine horn serving as the internal control and the contralateral horn assigned to either the injury model group or the TR-gel treatment group (Fig. 6A). At 7 days postcoitum, the number of embryo implantation sites was significantly lower in the model group than in the sham group. By contrast, the TR-gel group showed a significant recovery in implantation sites, reaching up to 62.5% of the level observed in the sham group (Fig. 6B). This restorative effect was further reflected in live birth rates at gestational day 21. While the model group exhibited a pronounced reduction in litter size, TR-gel treatment substantially increased the number of viable pups. Offspring in the TR-gel group also displayed normal growth throughout a 14-day postnatal observation period (Fig. 6C), confirming that the regenerated endometrium was capable of supporting full fetal development.

To evaluate potential effects on mid-gestational embryogenesis, we compared the PBS and TR-gel groups at 14 days postcoitum. No significant difference in embryo number was observed between the two groups (Fig. 6D), and histological examination revealed normal embryonic morphology with no evidence of developmental abnormalities (Fig. 6E). Together, these findings demonstrate that the TR-gel not only acts as a physical barrier to avert adhesion reformation but also mitigates oxidative stress and inflammation, thereby establishing a regenerative endometrial microenvironment conducive to the full recovery of fertility.

## DISCUSSION

Endometrial injury predominantly stems from iatrogenic uterine instrumentation or postoperative infection. These insults can reach the basalis layer and its microvascular plexus, triggering a persistent inflammatory cascade. Histopathologically, the lesion is characterized by impaired reconstitution of the endometrial stromal and epithelial compartments, a significant reduction in glandular density, and a notable decline in endometrial receptivity. Collectively, these alterations undermine the apposition, adhesion, and subsequent invasion of the blastocyst, ultimately resulting in female infertility or recurrent pregnancy loss (*45*, *46*). To tackle endometrial defects, the development of therapeutic materials that integrate biological activity with structural compatibility to native tissues is imperative.

In this study, we developed an injectable, thiol-rich immunomodulatory hydrogel (TR-gel) that effectively promotes endometrial regeneration and prevents IUAs. The high density of thiol moieties confers potent antioxidant and immunomodulatory activity, while the tunable mechanical properties closely match the elastic modulus of native endometrial tissue. The hydrogel forms a stable, in situ cross-linked network via simple mixing and injection, enabling minimally invasive intrauterine application. Moreover, the TR-gel remained well attached to the tissue surface, maintaining stable contact during deformation. This favorable tissue affinity can be attributed to two synergistic mechanisms. First, TR-gel is injectable and capable of in situ gelation, allowing it to conform closely to the irregular injured endometrial surface and form an intimate physical interface after administration. Second, the HA-based network can interact noncovalently with the tissue surface through hydrogen bonding and other interfacial interactions. In addition, the residual free thiol groups preserved in the thiol-rich network may further enhance local tissue attachment through thiol-disulfide exchange with tissue-associated proteins. Notably, this adhesive interaction is confined to the injured surface and does not translate into cross-tissue bridging. Instead, the TR-gel functions as a physical antiadhesion barrier: Its HA-based, non–cell-adhesive network prevents fibroblast infiltration and fibrous tissue connection between opposing wound surfaces, thereby blocking pathological readhesion. Additionally, the TR-gel has a porous structure that enables rapid yet stable swelling and an enzyme-responsive degradation profile. This controlled behavior not only ensures temporary mechanical integrity but also allows timely resorption, thereby reducing the risk of endometrial compression from undegraded material and supporting long-term tissue regeneration by synchronizing degradation with the healing process. Mechanistic studies revealed that the TR-gel scavenges ROS, inhibits NF-κB activation, and promotes M2 macrophage polarization, collectively mitigating inflammation and fibrosis.

Accordingly, we established an in vivo endometrial injury model in SD rats and treated them with intrauterine injections of TR-gel. The results of in vivo experiments demonstrated that the TR-gel could restore endometrial architecture, suppress collagen deposition, and up-regulate genes involved in angiogenesis and cellular proliferation. The therapeutic underpinning of TR-gel resides in its exceptionally high thiol density. Cumulative evidence demonstrates that thiol moieties confer robust antioxidative and anti-inflammatory bioactivities that orchestrate multicellular repair cascades. This chemistry provides a clinically translatable, universal platform for precision regeneration of mechanically fragile and oxidatively stressed tissues, including, but not limited to, the endometrium, myocardium, spinal cord, and bile duct (*47*–*49*). Whereas intrauterine instillation in the murine model necessitated a transabdominal approach, the TR-gel formulation is readily adaptable to a fully ambulatory, nonsurgical paradigm in humans. A flexible soft-tipped catheter can be advanced transcervically under continuous transvaginal ultrasound guidance, permitting atraumatic deposition of the hydrogel within the uterine cavity without mechanical disruption of the nascent endometrium. This seamless translational pathway positions intracavitary TR-gel delivery as a safe, efficacious, and minimally invasive intervention for patients with endometrial injury, with immediate potential for future clinical evaluation.

However, the underlying mechanism of endometrial reconstruction remained unclear. In a rat model of endometrial injury, we used RNA sequencing and immunofluorescence staining to explore the therapeutic mechanism of the TR-gel. The RNA sequencing results revealed significant changes in the expression profiles of genes related to cell proliferation, angiogenesis, and ECM remodeling in the endometrial tissues treated with TR-gel compared to the control group, which are known to play crucial roles in promoting endometrial regeneration and repair. Further mechanistic investigation demonstrated that, during endometrial repair, the TR-gel effectively induces M2 macrophage polarization and suppresses the release of proinflammatory cytokines. Specifically, levels of TNF-α and IL-1β were down-regulated, along with a reduction in TGF-β1, a cytokine that promotes fibroblast activation and proliferation. These findings suggest that the advantageous microenvironment created by the abundant TR-gel in the uterine cavity can inhibit excessive fibrogenesis through its anti-inflammatory effects. Thus, the TR-gel may modulate the local immune microenvironment to facilitate tissue healing.

Following endometrial injury, successful repair critically depends on the rapid reestablishment of compromised blood supply. CD34, a heavily glycosylated transmembrane protein, is specifically expressed on vascular endothelial progenitor cells and mature endothelial cells (*50*). Our study demonstrates that TR-gel treatment significantly up-regulates CD34 expression and increases the density of CD34-positive vessels in the endometrium. This indicates that the anti-inflammatory and proregenerative microenvironment created by TR-gel effectively activates and supports the function of CD34-positive endothelial cells, leading to the reconstruction of a dense and uniformly distributed functional microvascular network, thereby promoting both structural and functional regeneration of the endometrium.

The process of embryo implantation is complex and orchestrated by multiple factors. Successful implantation requires high-quality embryos, optimal endometrial receptivity, and precise temporal coordination between embryonic and endometrial development. Endometrial receptivity is regulated by a variety of elements that induce molecular and structural modifications within the uterine milieu, thereby creating a supportive niche for blastocyst implantation (*51*). Beyond facilitating endometrial repair, the application of the TR-gel has been shown to further enhance endometrial receptivity, thus establishing a favorable microenvironment for subsequent embryo implantation and successful offspring production.

As the primary objective of this study was to establish a proof of concept for an injectable, immunomodulatory hydrogel targeting IUA, we focused on a standardized rat endometrial injury model and a single bioactive mechanism centered on thiol-mediated redox regulation and macrophage polarization. While this reductionist approach successfully delineated the core mode of action, it inherently does not capture the multifactorial complexity of clinical IUA, which often involves chronic inflammation, secondary infection, severe compromise of the basal layer, and heterogeneous tissue responses. To bridge this translational gap, future studies must validate the TR-gel in more physiologically and pathologically relevant models, such as those incorporating bacterial coinfection, repetitive injury, or ischemic conditions. Furthermore, the modular design of the hydrogel platform invites the development of combination formulations, for example, through the integration of exogenous stem cells, targeted antifibrotic agents, or sustained release angiogenic factors, to address the diverse etiologies of human IUA. Advancing these strategies will be critical for tailoring this promising immunomodulatory material to the nuanced realities of clinical disease.

Although this study has yielded encouraging results, several aspects warrant further improvement and investigation. The rat model of IUA used in this study was induced by mechanical injury. By simulating the aberrant repair process following damage to the endometrial basalis layer, this model adequately recapitulates key pathological features such as fibrotic progression, glandular reduction, and endometrial thinning. It is therefore widely used as an important animal model for exploring the pathogenesis of IUA, evaluating endometrial repair strategies, and screening potential therapeutic interventions. Nevertheless, we fully acknowledge the inherent species-specific limitations of this model. Rats exhibit an estrous cycle, and the mechanisms of endometrial repair in rodents differ substantially from those in humans, who have a true menstrual cycle. This physiological discrepancy may limit the interpretation of the regenerative processes observed in this study and constrain the prediction of its clinical translational potential.

Based on these considerations, to further enhance the translational value of the TR-gel, future studies should systematically validate its efficacy in animal models that more closely recapitulate human physiology. Wang *et al.* (*1*) successfully established an IUA model in the nonhuman primate rhesus monkey and demonstrated that a HA gel loaded with human umbilical cord-derived mesenchymal stem cells effectively promoted endometrial regeneration, reduced fibrosis, and restored menstrual cycles, thereby providing crucial support for clinical translation. Consequently, priority should be given to evaluating the long-term safety and efficacy of the TR-gel in nonhuman primates with true menstrual cycles, such as rhesus or cynomolgus monkeys. In addition, the establishment of a humanized endometrial organoid-immune cell coculture system would serve as an important complementary platform to further elucidate the interactions between the TR-gel and the human endometrial microenvironment, thereby enabling a multidimensional validation of its repair mechanisms.

In conclusion, this study has demonstrated that the TR-gel not only offers a potentially viable therapeutic option for the treatment of IUA but also, more crucially, establishes a generalizable design strategy for attaining functional regeneration of complex tissues. Future efforts should continue to be directed toward promoting the clinical application of the TR-gel platform.

## MATERIALS AND METHODS

### Preparation and characterization of the hydrogel

HA-A and HA-SH were purchased from Blafar Ltd. Injectable hydrogels were formed by mixing HA-A and HA-SH solutions in 0.1 M Na_2_HPO_4_ buffer (hydrogel at pH 7.4). Briefly, the substitution degrees of HA-A and HA-SH were 7 and 35% respectively, as provided by the supplier. To optimize the mechanical properties of the hydrogel for adaptation to the endometrial microenvironment, three total polymer concentrations (1, 2, and 3%) were examined, and the volume ratios of HA-A to HA-SH were set at 4:1, 5:1, and 6:1. These corresponded to acrylate-to-thiol molar ratios of 1:1.25, 1:1, and 1.2:1, respectively.

Rheological properties were measured using a parallel-plate rheometer. Gelation kinetics were monitored by time-sweep tests (1% strain, 1 Hz). Shear-thinning behavior was assessed by sweeping shear rates from 0.1 to 100 s^−1^. One day before the test, 600 μl of mixed solution was incubated thoroughly in a flat cylindrical mold. The intact hydrogel was then loaded onto parallel plates, and measurements were performed at a frequency of 1 Hz with a strain amplitude of 1% to monitor the storage modulus (*G*′) and loss modulus (*G*″). After each time sweep, angular frequency sweep tests were conducted over a frequency range of 0.1 to 10 rad/s at a constant strain of 1% to evaluate frequency-dependent moduli. Subsequently, strain sweep tests were carried out over a strain range of 0.1 to 1000% at a fixed frequency of 1 Hz. The critical strain value was defined as the crossover point at which the storage modulus equaled the loss modulus. Microstructure was imaged by SEM after lyophilization.

The thiol content of the hydrogel was determined using Ellman’s reagent [5,5′-dithiobis-(2-nitrobenzoic acid)] (Aladdin, China). All measurements were carried out in a reaction buffer containing 0.1 M Na_2_HPO_4_ and 1 mM EDTA (pH 8.0). A 30 mM l-cysteine hydrochloride monohydrate stock solution was prepared in the reaction buffer and serially diluted to final concentrations of 0, 0.5, 1.0, 1.5, and 2.0 mM. Ellman’s reagent was freshly dissolved in the reaction buffer at 10 mM. For each assay, 50 μL of Ellman’s solution was mixed with 1 mL of reaction buffer, followed by the addition of a 240-μl hydrogel construct at 0 hours, 4 hours, 8 hours, 1 day, 3 days, and 7 days after gelation. After 12 hours of incubation at 4°C, absorbance was measured at 412 nm. The thiol content of the hydrogel was calculated on the basis of the standard curve constructed using the l-cysteine standards. All measurements were performed in triplicate.

Hydrogel samples were incubated in PBS medium containing 0, 0.1, 1, 10, and 100 U/ml hyaluronidase at 37°C under agitation. The weight of the hydrogel (Wt) was recorded at each time interval after the removal of medium. The relative mass percentage was calculated as Wt/*W*_0_ × 100%, where *W*_0_ represents the initial weight of the hydrogel.

### Ex vivo tissue adhesion

Fresh rat uterine tissue was coated with TR-gel precursor. After 10 min of gelation, hydrogel retention was assessed visually during tissue manipulation.

### Cytotoxicity assessment

A CCK-8 assay was used to evaluate the cytocompatibility of the TR-gel. First, TR-gels were prepared under sterile conditions and immersed in Dulbecco’s modified Eagle’s medium (DMEM)/F12 medium (0.1 g/ml) at 37°C for 24 hours and filtered through a 0.22-μm membrane to obtain the extraction medium. HUVECs were seeded at 5000 cells per well and cultured in the extraction medium for 1, 3, and 5 days, with cells in normal medium serving as the control. At each time point, the medium was replaced with fresh medium containing 10% CCK-8 reagent, followed by incubation for 2 hours at 37°C. Absorbance was measured at 450 nm using a microplate reader. Cell viability was calculated as

Viability(%)=[OD(sample)/OD(control)]×100%

### Wound scratch assay

HUVECs were seeded in six-well plates at 1 × 10^5^ cells per well and grown to 90% confluency. A scratch was introduced using a 200-μl pipette tip, and detached cells were removed by washing with PBS. The cells were then incubated with TR-gel extraction medium. Images were acquired at 0 and 24 hours using an Olympus IX81 microscope, and wound closure was quantified with ImageJ software. Five replicates were analyzed per group.

### Cell migration and invasion assays

Cell migration and invasion were assessed using Transwell assays. For the migration assay, 4 × 10^4^ HUVECs were seeded in serum-free medium in the upper chamber. For the invasion assay, 3 × 10^5^ HUVECs were seeded in Matrigel-coated upper chambers. In both assays, the lower chamber contained medium supplemented with 200 μl of hydrogel. After 24 hours of incubation, cells that had migrated or invaded to the lower surface were fixed with 4% paraformaldehyde, stained with crystal violet, and imaged under an Olympus microscope.

### Tube formation assay

Each well of a 96-well plate was coated with 50 μl of Matrigel and polymerized at 37°C for 30 min. HUVECs (3 × 10^4^ per well) were seeded in 100 μl of TR-gel extraction medium, with plain DMEM as a control. After 4 hours of culture, tube-like structures were imaged, and the number of tubes per field was quantified using ImageJ. Three replicates were included per group.

### Intracellular antioxidant activity

RAW 264.7 macrophages (1 × 10^4^ cells per well) were seeded onto the lower chamber of 48-well plates and cultured overnight, and 200 μl of different hydrogel samples was added to the upper chamber of a Transwell assay filter. Subsequently, the cells were exposed to LPS (1 μg/ml) to establish an oxidative stress model. After treatment, ROS levels were assessed using the fluorescent probe DCFH-DA according to the manufacturer’s instructions. Briefly, cells were incubated with 10 μM DCFH-DA at 37°C for 20 min in the dark, followed by three gentle washes with PBS. Fluorescence images were captured using a fluorescence microscope to evaluate intracellular ROS accumulation and cell viability.

### NF-κB immunofluorescence staining and confocal microscopy

Briefly, sterile coverslips of appropriate size were placed into the wells of a six-well plate, and well-conditioned RAW264.7 cells were seeded onto the coverslips. After the cells had adhered, 200 μl of different hydrogel samples was added to the upper chamber of a Transwell assay filter, followed by incubation in a cell culture incubator for 48 hours. After the incubation, aspirate the culture medium and wash once with PBS. Fix the cells with 4% paraformaldehyde at room temperature for 30 min. Then, remove the fixative and wash the cells three times with PBS. After being exposed to immunoblocking antibodies, cells were incubated with phosphorylated NF-κB/p65 primary antibody (ABclonal, China) at 4°C overnight following cell fixation and blocking. Macrophages were incubated with Cy3-conjugated secondary antibody for 1 hour at room temperature. Cells were stained with 4′,6-diamidino-2-phenylindole (DAPI) for 5 min at room temperature, washed, and then visualized by a confocal microscope.

### Macrophage polarization assay

RAW264.7 cells were inoculated into six-well culture plates with aseptic cover slides and treated with corresponding extraction medium for 24 hours, which included the following groups: LPS [induced by LPS (1 μg/ml)], LPS and HA-A, LPS and HA-SH, and LPS and TR-gel. After rinsing in PBS, the cells were fixed in 4% paraformaldehyde for 30 min, rinsed three times for 5 min with PBS, and blocked with 5% bovine serum albumin for 1 hour. Then, the primary antibody was added, and the cells were incubated overnight at 4°C. The cells were then rinsed three times for 5 min in PBS with Tween and incubated with a fluorescent secondary antibody according to the TSA Plus Kit manufacturer’s protocols. The cells were stained with DAPI and then sealed with antifade mounting medium and observed under a laser scanning confocal microscope.

### In vivo IUA model construction and treatment

Eight-week-old sexually mature female Sprague-Dawley rats (specific pathogen–free grade, 220 to 250 g) were obtained from the Shanghai Experimental Animal Center. All experimental procedures were conducted in accordance with the institutional guidelines for the care and use of laboratory animals at Xuzhou Medical University and approved by the Animal Ethics Committee (no. 202509 T005). Animals were housed in a facility with controlled temperature (20° to 26°C) and humidity (40 to 70%) under a 12-hour light/dark cycle (lights on at 6:00 a.m.), and provided with standard sterile diet and water ad libitum.

A total of 111 rats (96 female rats and 15 male rats) were used in this study. These included 21 females for biosafety evaluation, 15 females for assessment of hydrogel-mediated endometrial repair, 15 females for transcriptomic sequencing, 15 females for HYP assay, and 30 females and 15 males for fertility analysis. Vaginal cytology was performed before experiments to synchronize the estrous cycle.

An endometrial injury model was established in 8-week-old female SD rats as previously described (*52*). The surgical procedure for the animal model was recorded in fig. S5. At diestrus, rats were anesthetized with tribromoethanol (0.2 ml/10 g, Aibei Biotechnology), and a 2-cm vertical incision was made in the abdominal wall to identify the right uterine horn. Then, a mini-endometrial curette was inserted into the uterine cavity to scrape the endometrium several times until the uterine cavity walls became rough and congested. The left uterine horn of each rat was left untreated as a self-control. Animals were randomly allocated into three groups: (i) the sham group, with abdominal incision only and no uterine injury; (ii) the model group, the right uterine horn was injured and 200 μl of PBS was injected; and (iii) the TR-gel group, in which the IUA model was established and 200 μl of TR-gel was injected into the right uterine horn. The uterine horn was then sutured and returned to the abdominal cavity. The muscle layer and skin were closed using 4-0 absorbable sutures.

### Ex vivo hemolysis test

Fresh whole blood (2 ml) collected from a Sprague-Dawley rat was diluted with 20 ml of normal saline and centrifuged at 1200*g* for 15 min. The supernatant was discarded, and the erythrocyte pellet was washed three times with saline before being resuspended to obtain a 2% (v/v) red blood cell (RBC) suspension. For the test, 0.5 ml of TR-gel extraction medium was mixed with 0.5 ml of the RBC suspension as the experimental group (W1). Positive and negative controls were prepared by mixing 0.5 ml of RBC suspension with 0.5 ml of deionized water (W0) or 0.5 ml of saline (W2), respectively. All samples were incubated at 37°C for 1.5 hours and then centrifuged, and the absorbance of the supernatant was measured at 540 nm using a microplate reader. The hemolysis rate was calculated as follows

Hemolysis rate(%)=(W1−W2)/(W0−W2)×100

### In vivo degradation and biocompatibility of TR-gel

To assess in vivo degradation and biocompatibility, 200 μl of TR-gel was injected subcutaneously into the dorsal region of each rat. The implants and surrounding tissues were harvested on days 0, 14, and 28 postinjection for evaluation of hydrogel degradation and tissue response. Specimens were sectioned and subjected to H&E staining for histopathological evaluation, as well as immunohistochemical staining for anti–α-SMA (1:500; GB12045, Servicebio) and anti–TNF-α (1:100; ER65189, Huabio). For safety assessment, rats were randomly assigned to two groups (*n* = 3 per group): (i) PBS group, receiving 200 μl of PBS injected into the uterine lumen; and (ii) TR-gel group, receiving 200 μl of TR-gel injected into the uterine lumen. On day 14, blood samples and major organs (brain, heart, liver, uterus, lung, and kidney) were collected for safety evaluation.

### Histology and immunofluorescence

Uterine tissues were fixed, dehydrated, embedded in paraffin, and sectioned at 4-μm-thickness. H&E staining was used to evaluate endometrial thickness and gland count, whereas Masson’s trichrome staining was used to assess collagen deposition and fibrosis. Stained sections were imaged and analyzed using ImageJ software.

The HYP content in rat uterine tissue was measured using a commercial assay kit (Solarbio, China). Approximately 0.2 g of uterine tissue was accurately weighed, placed into an EP tube, and thoroughly minced to facilitate subsequent digestion. Then, 2 ml of extraction solution was added, and the mixture was digested in a boiling water bath for 2 to 6 hours until no visible large tissue clumps remained. After cooling, the pH was adjusted to 6 to 8 using ∼1 ml of 10 M NaOH solution, and the volume was brought to 4 ml with distilled water. The mixture was centrifuged at 16,000 rpm for 20 min at 25°C. One milliliter of the supernatant was collected, and its absorbance [optical density (OD) value] was measured at 560 nm using distilled water as a blank. The HYP content was calculated on the basis of the measured OD value using the corresponding formula.

For immunofluorescence staining, serial sections were immuno-labeled with anti-CD34 (1:1000; AB182981, Abcam), anti–IL-1β (1:100; AF5103, Affinity), anti–TGF-β1 (1:500; GB12045, Servicebio), anti–TNF-α (1:100; ER65189, Huabio), anti-CD206 (1:1000; GB13428, Service), and anti-CD80 (1:1000; GB13428, Service) overnight at 4°C. Then, these sections were incubated with appropriate fluorophore-conjugated secondary antibodies (Invitrogen, USA, 1:200). Nuclei were counterstained with DAPI (20 μg/ml) for 5 min, and images were acquired using a fluorescence microscope.

### RNA sequencing and analysis

To investigate the molecular mechanisms underlying the therapeutic effect of the TR-gel on endometrial regeneration, endometrial tissues were collected 14 days postsurgery from three experimental groups: sham-operated, model (endometrial injury without treatment), and TR-gel (endometrial injury treated with TR-gel). Total RNA was extracted from uterine tissues using TRNzol Universal Reagent (TIANGEN, DP424). cDNA libraries were constructed with the NEBNext Ultra RNA Library Prep Kit for Illumina (New England Biolabs, E7530) and sequenced on an Illumina NovaSeq 6000 platform (150–base pair paired-end reads; Novogene, Beijing).

DEGs were identified using thresholds of |log_2_(fold change)| > 1 and adjusted *P* value < 0.05. DEG distributions were visualized via volcano plots and heatmaps. Functional annotation of DEGs was performed through GO enrichment and gene set enrichment analysis to identify biologically relevant pathways and processes.

### Fertility assessment

Fourteen days postsurgery, 30 female SD rats were paired with sexually mature male rats at a 2:1 ratio. The observation of a vaginal plug was designated as gestational day (GD) 1. To evaluate reproductive outcomes, five rats per group were monitored for live births during GD 21 to 23. The resulting offspring were nurtured for an additional 14 days to screen for developmental abnormalities. Embryonic morphology was examined histologically by H&E staining.

### Statistical analysis

Data are presented as means ± SD. Comparisons between two groups were performed using unpaired Student’s *t* test, while multiple group comparisons were conducted by one-way analysis of variance (ANOVA) with appropriate post hoc tests. Significance levels are indicated as *P < 0.05, **P < 0.01, ***P < 0.001, and ****P < 0.0001. All image-based quantifications, including size measurements and fluorescence intensity analyses, were performed using ImageJ software (National Institutes of Health, USA). GraphPad Prism 10 was used for all statistical evaluations.
