# Supplementary material for: A thiol-rich immunomodulatory injectable hydrogel suppresses endometrial fibrosis to restore fertilization
Source: Sci Adv. 2026 Aug 12;12(33):eaef5617. doi: 10.1126/sciadv.aef5617 (PMC13464475; doi:10.1126/sciadv.aef5617)
Supplement: Supplementary file 1 [file sciadv.aef5617_sm.pdf]

Science Advances
AAAS

Supplementary Materials for

A thiol-rich immunomodulatory injectable hydrogel suppresses endometrial fibrosis to restore fertilization

Mingwei Li *et al.*

Corresponding author: Qiang Li, liq@xzhmu.edu.cn; Rijian Song, rijian.song@ucd.ie; Wenxin Wang, wenxin.wang@ucd.ie

Sci. Adv. **12**, eaef5617 (2026)
DOI: 10.1126/sciadv.aef5617

This PDF file includes:

Figs. S1 to S8

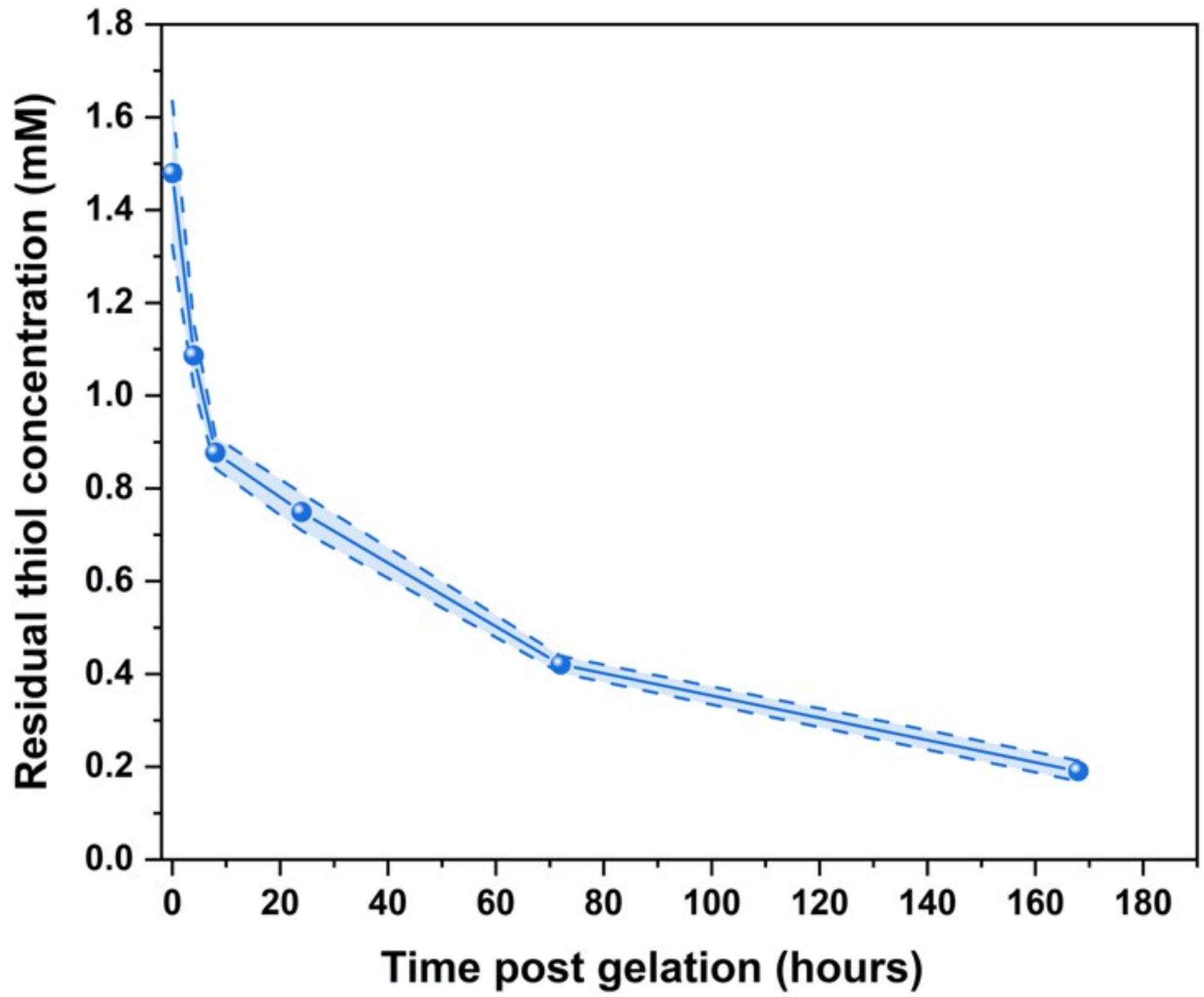

Fig. S1. Residual thiol concentration in the TR-gel. Data are presented as mean ± SD (n = 3).

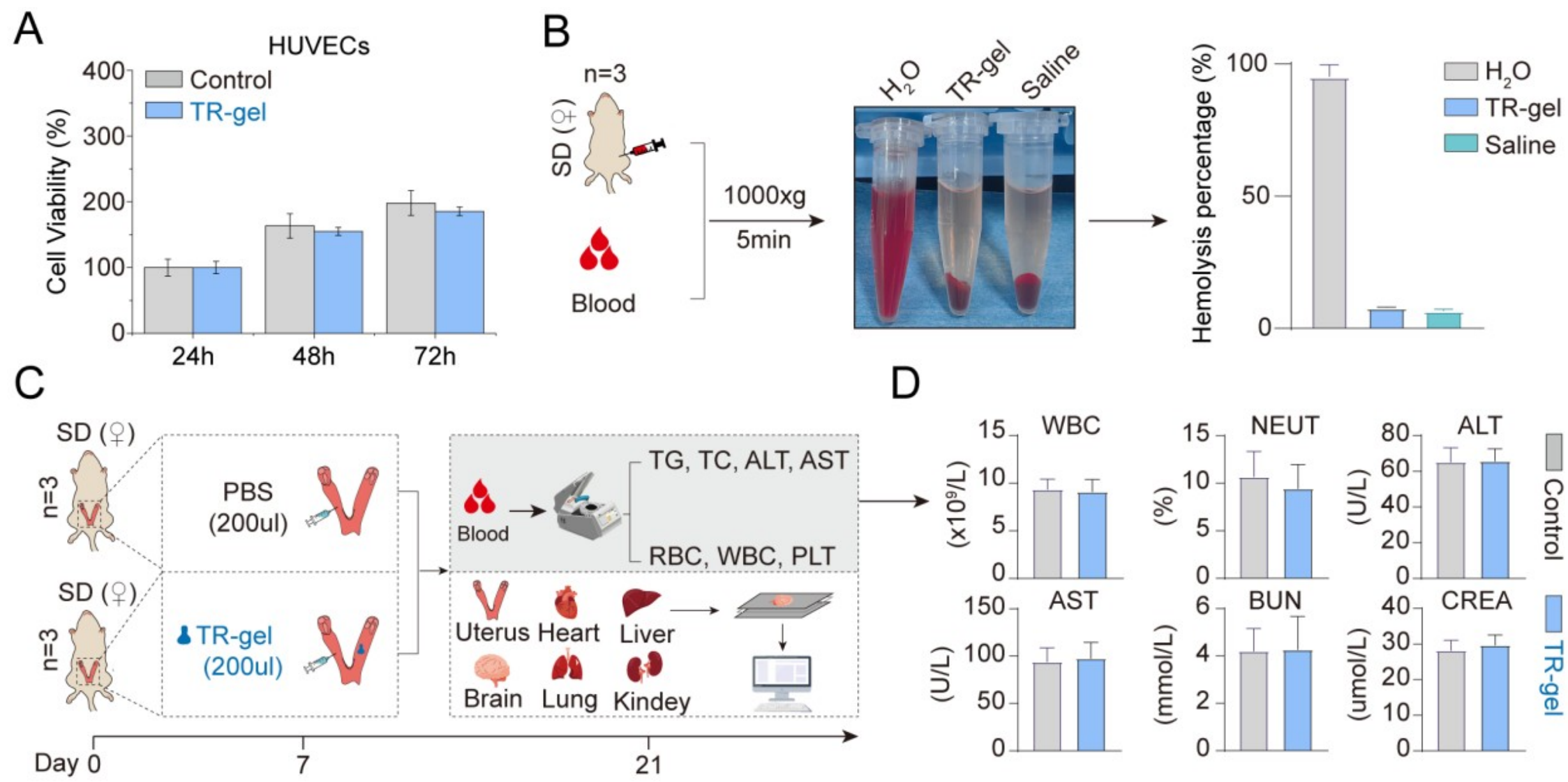

Fig. S2. *In vitro* and *in vivo* biocompatibility evaluation of the TR-gel. (A) Endothelial cell viability assay. Human umbilical vein endothelial cells (HUVECs) were cultured with the TR-gel extract for 24, 48, and 72 hours. Cell viability was assessed using the CCK-8 assay and normalized to the control group (culture medium only). **(B)** Representative images of erythrocytes after incubation with deionized water (positive control), saline (negative control), and TR-gel extract. The hemolysis rate of the TR-gel extract was negligible and remained significantly below the 5% ISO safety threshold. **(C)** Schematic of the systemic biosafety assessment in a rat model. Female Sprague-Dawley (SD) rats were administered a single injection of either PBS (control) or TR-gel. Blood samples and major organs (brain, heart, liver, lung, kidney, and uterus) were collected on day 14 after treatment for hematological and histological analysis. **(D)** Systemic toxicity assessment: hematological parameters (WBC and NEUT) and serum biochemistry markers (ALT, AST, BUN, and CREA). Data are presented as mean ± SD (n = 3). (Two-way ANOVA with Bonferroni's post hoc test).

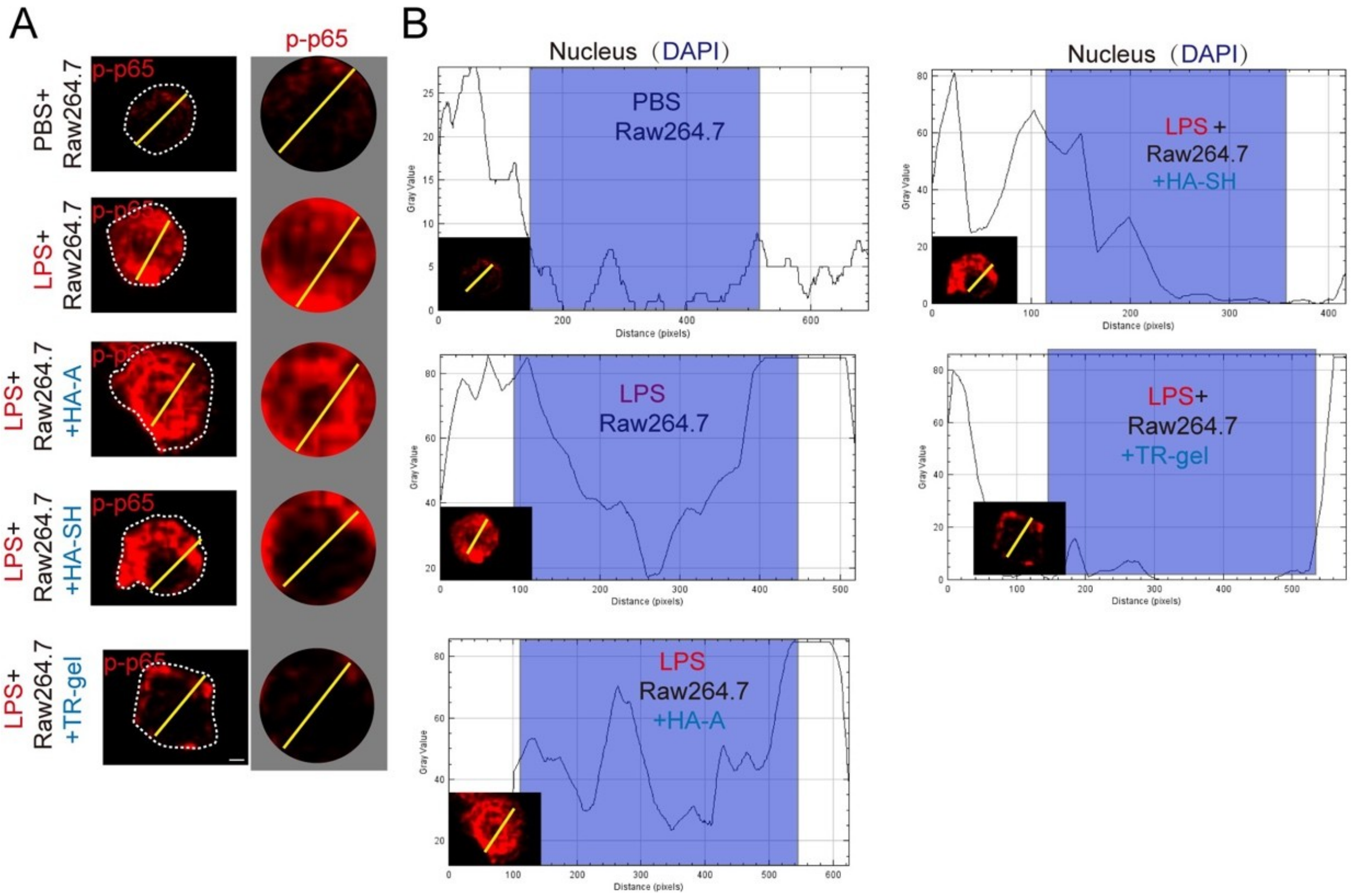

Fig. S3. Quantitative analysis of p-p65 nuclear translocation across the RAW 264.7 cell area in Fig. 4G. (A) The cell area (indicated by white dashed lines) and ROI (yellow straight lines) used in the fluorescent intensity analysis. **(B)** p-p65 fluorescence intensity distribution measured by grey value across the ROI. The blue shaded region in the histogram indicates the nuclear area. The reuse is intentional and reflects analysis of the same image dataset.

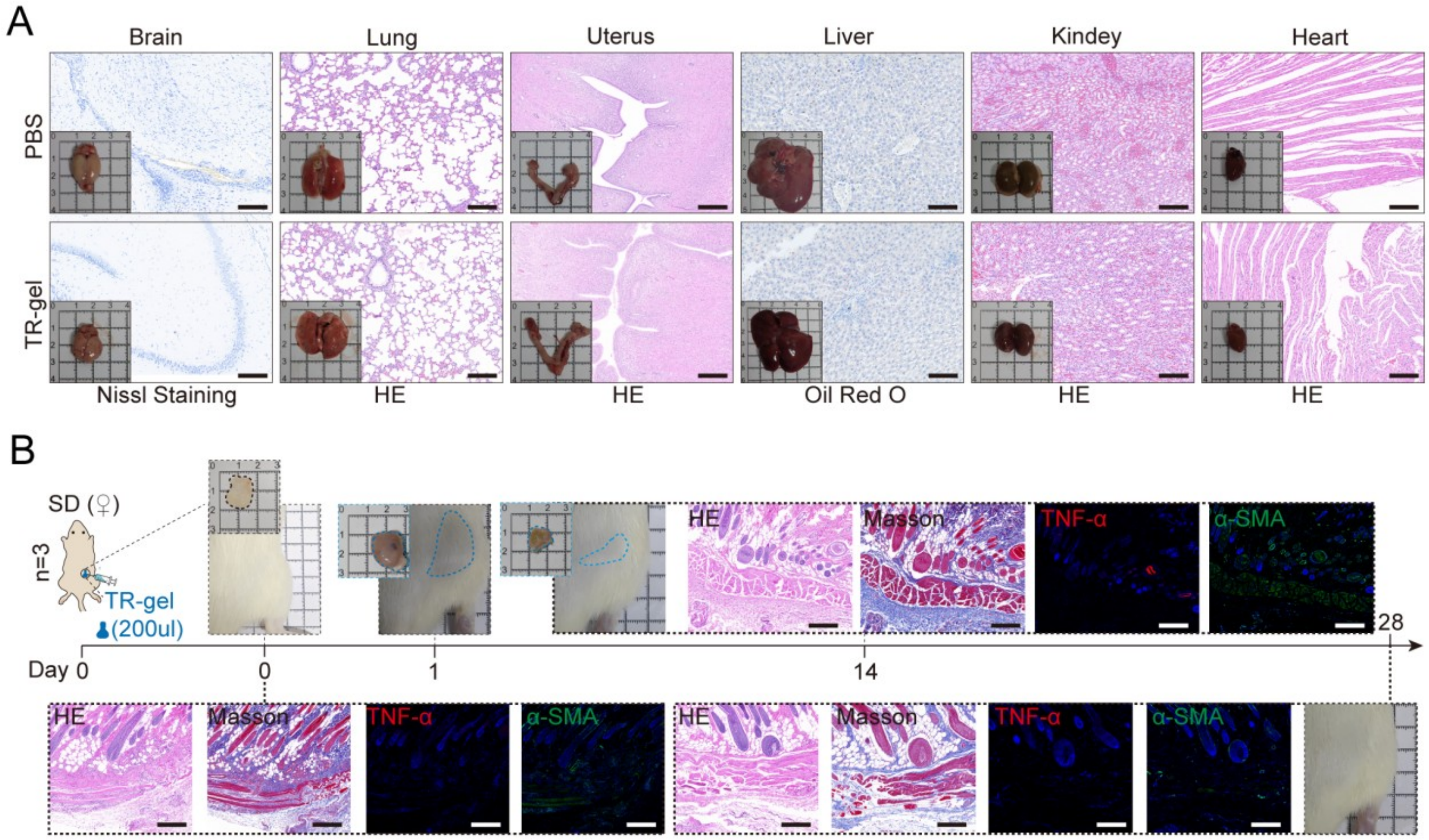

Fig. S4. ***In vivo*** **degradation and local biocompatibility of the TR-gel. (A)** Histopathological assessment of major organs. Representative images of hematoxylin and eosin (H&E) staining for the heart, liver, spleen, lung, uterus, and kidney; Nissl staining for the brain; and Oil Red O staining for the liver, collected from rats 14 days after injection of phosphate-buffered saline (PBS) or thiol-rich immunomodulatory hydrogel (TR-gel). Scale bars = 200 μm. **(B)** Local tissue response and degradation profile. Histological evaluation of the implantation site and surrounding tissues at post-operative days (PODs) 14 and 28 showed that the TR-gel degraded progressively over time. From left to right: H&E staining showing overall tissue architecture and inflammatory cell infiltration; Masson's trichrome staining highlighting collagen deposition (blue); Immunofluorescent staining for the pro-inflammatory cytokine TNF-α and the myofibroblast marker α-SMA. Scale bars = 200 μm.

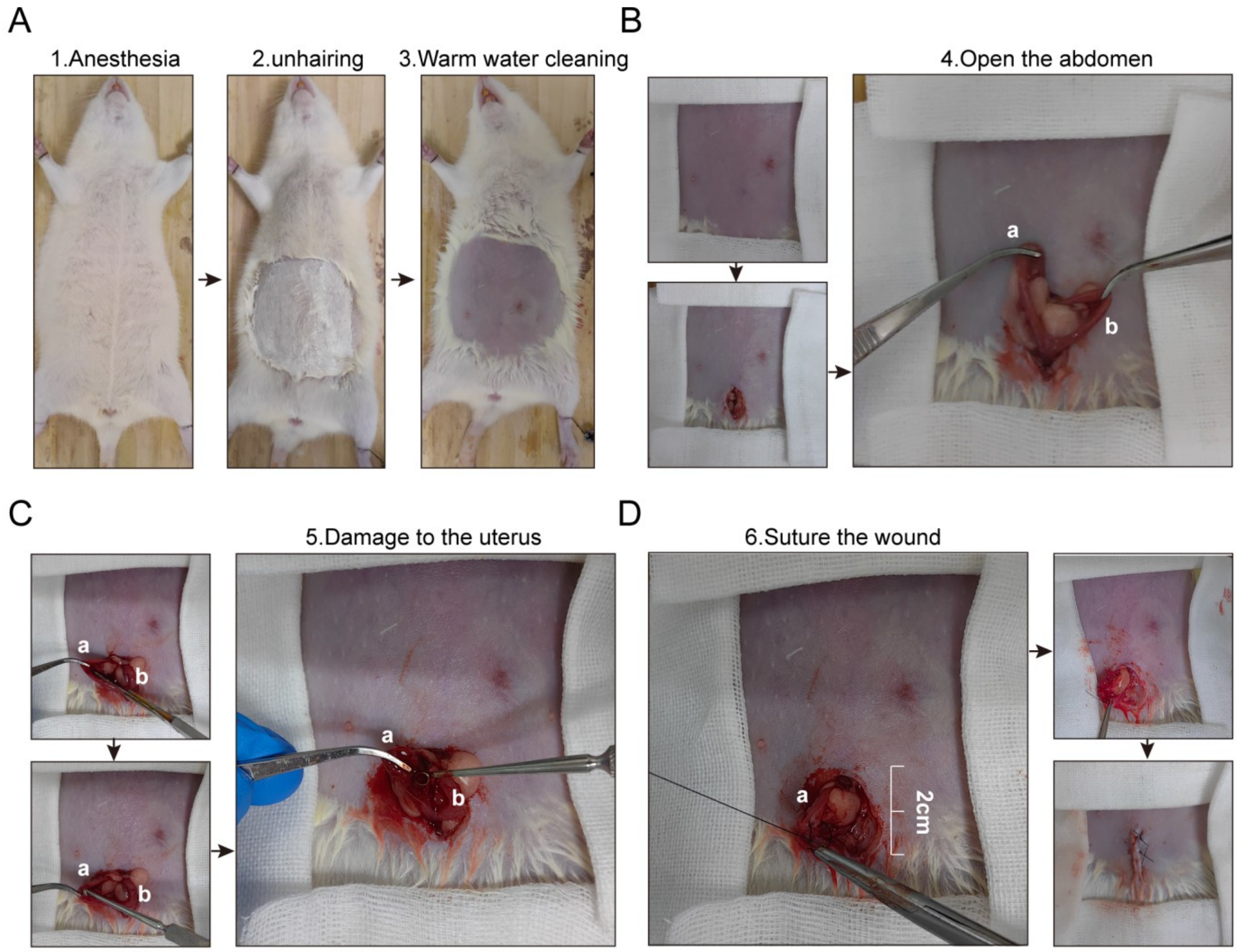

Fig. S5. Surgical procedure for establishing the rat intrauterine adhesion (IUA) model. (A) Preoperative preparation. Rats were anesthetized, and the abdominal area was shaved and cleansed with warm water. **(B)** Uterine exposure. A midline laparotomy was performed to expose the bilateral uterine horns. **(C)** Endometrial injury induction. The right uterine horn was mechanically abraded using a mini-endometrial curette inserted through the uterine lumen until the endometrial surface appeared rough and congested. The contralateral horn remained untouched as an internal control. **(D)** Wound closure. The uterine wall and abdominal incision were sutured sequentially.

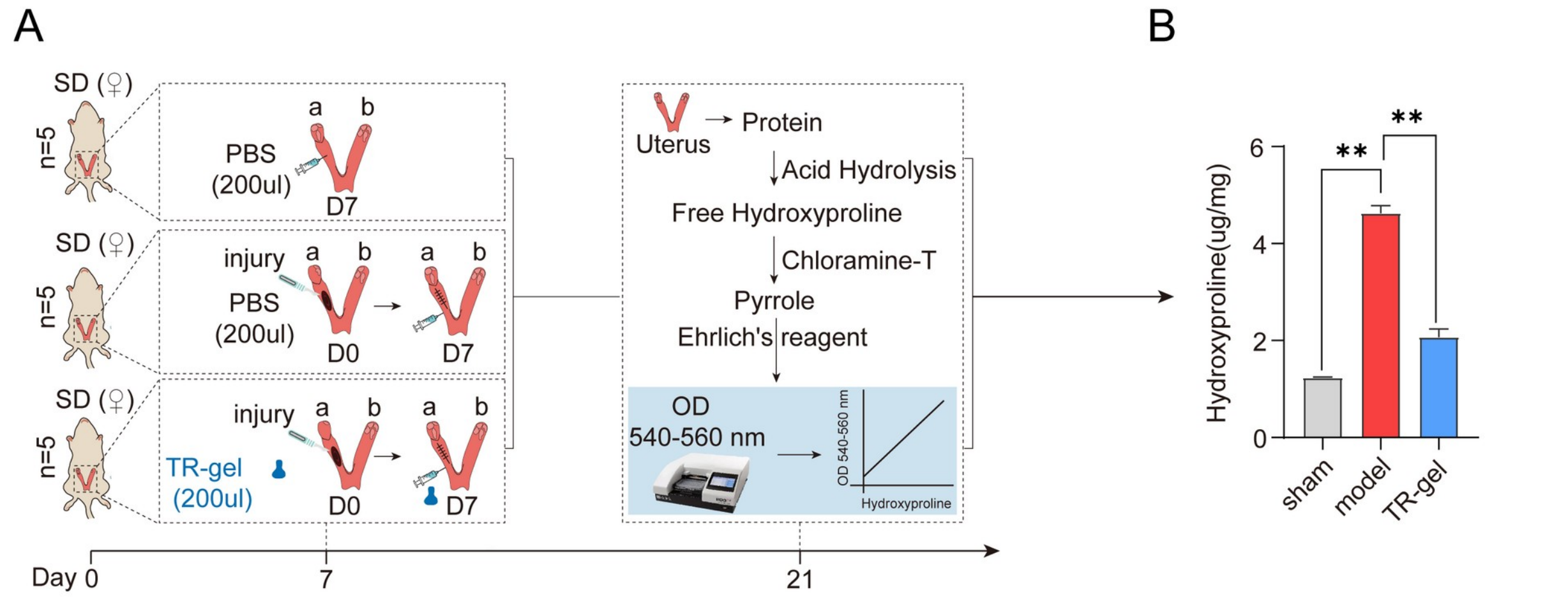

Fig. S6. Hydroxyproline (HYP) content in rat endometrial tissue. (A) Experimental timeline and assay procedure for HYP detection in each group. **(B)** Quantified HYP levels in rat endometrial tissue across groups. Data are presented as mean ± SD. *$p < 0.05$, **$p < 0.01$, ***$p < 0.001$, ****$p < 0.0001$ compared with the control group by unpaired t-test.

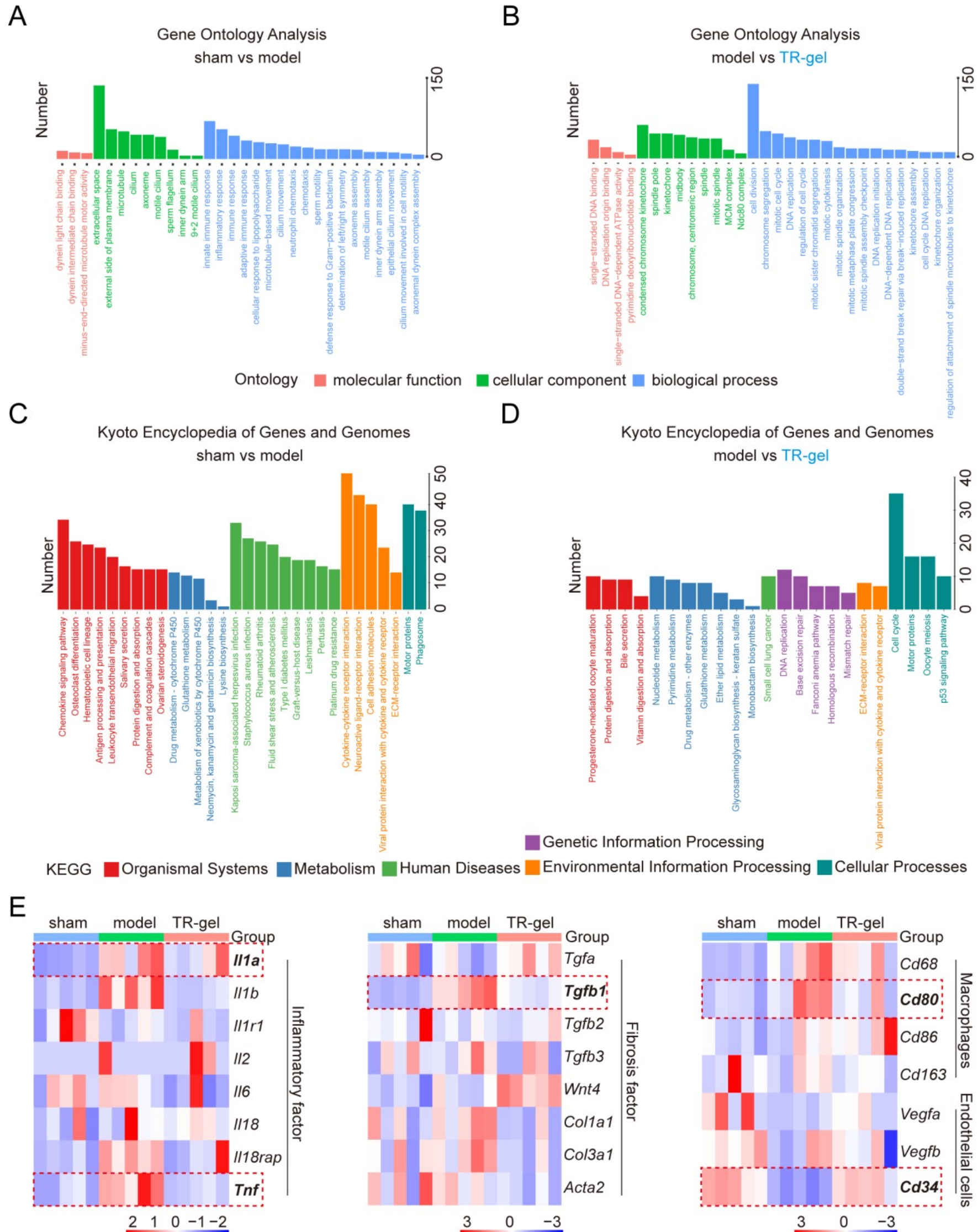

Fig. S7. Transcriptomic profiling of endometrial regeneration following TR-gel treatment. **(A, B)** Gene Ontology (GO) enrichment analysis of differentially expressed genes between the sham and model groups **(A)** and between the TR-gel and model groups **(B)**. **(C, D)** Kyoto Encyclopedia of Genes and Genomes (KEGG) pathway analysis of differentially expressed genes between the sham and model groups **(C)** and between the TR-gel and model groups **(D)**. **(E)** Heatmap depicting expression patterns of secretory factor genes associated with inflammatory and fibrotic pathways across the three experimental groups.

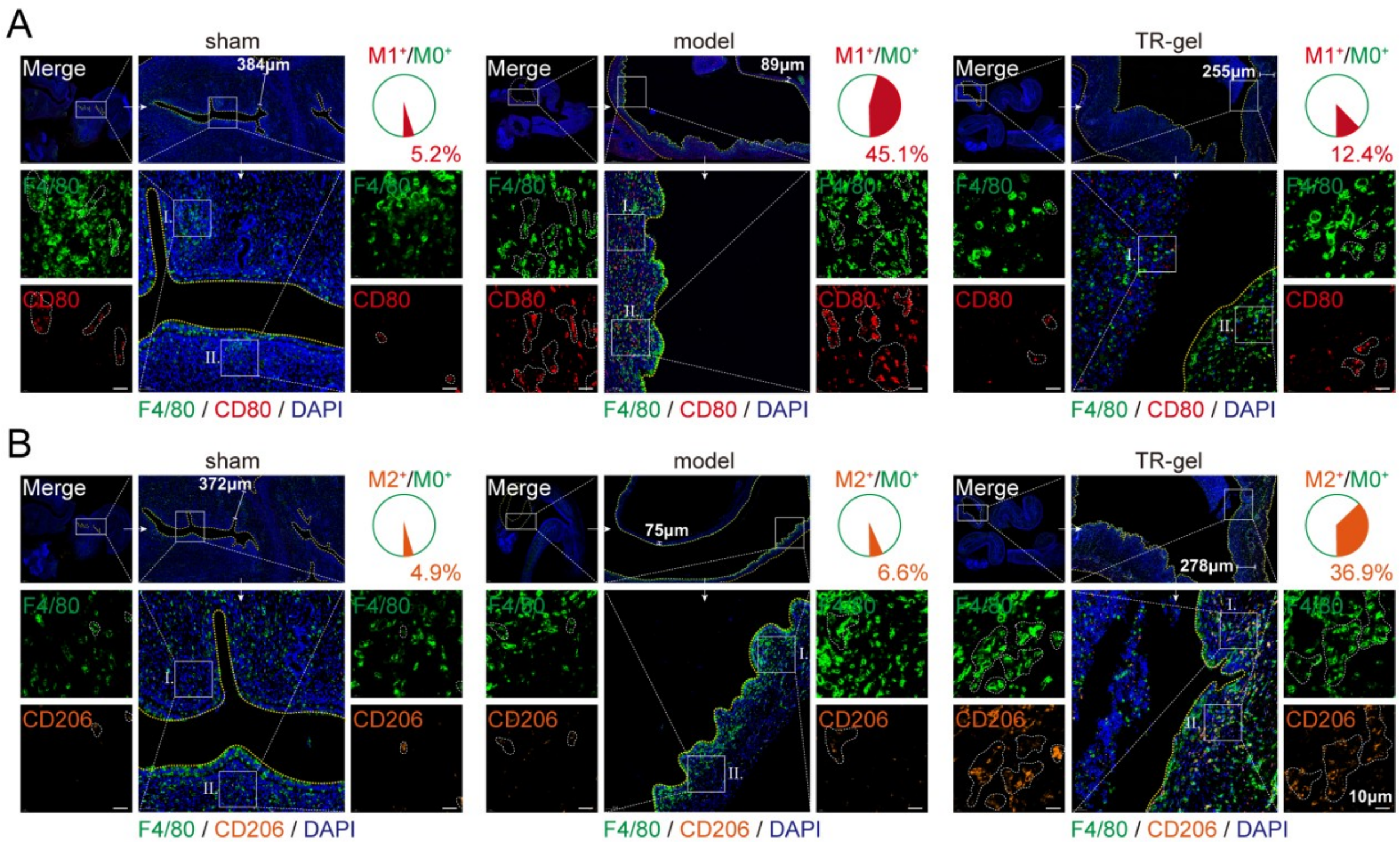

Fig. S8. TR-gel promotes macrophage recruitment and polarization *in vivo*. (A) Representative images showing co-localization of the M1 macrophage marker CD80 (red) with the pan-macrophage marker F4/80 (green); nuclei were counterstained with DAPI (blue). The percentages of CD80-positive cells in the sham, model, and TR-gel groups were 5.2%, 45.1%, and 12.4%, respectively. Scale bar = 10 µm. **(B)** Representative images showing co-localization of the M2 macrophage marker CD206 (orange) with F4/80 (green). The percentages of CD206-positive cells (M2 phenotype) in the sham, model, and TR-gel groups were 4.9%, 6.6%, and 36.9%, respectively. Scale bar = 10 µm. Magnified regions are indicated by white squares.
